# Calorie Restriction Increases Muscle Mitochondrial Biogenesis in Healthy Humans

**DOI:** 10.1371/journal.pmed.0040076

**Published:** 2007-03-06

**Authors:** Anthony E Civitarese, Stacy Carling, Leonie K Heilbronn, Mathew H Hulver, Barbara Ukropcova, Walter A Deutsch, Steven R Smith, Eric Ravussin

**Affiliations:** Pennington Biomedical Research Center, Baton Rouge, Louisiana, United States of America; Albert Einstein College of Medicine, United States of America

## Abstract

**Background:**

Caloric restriction without malnutrition extends life span in a range of organisms including insects and mammals and lowers free radical production by the mitochondria. However, the mechanism responsible for this adaptation are poorly understood.

**Methods and Findings:**

The current study was undertaken to examine muscle mitochondrial bioenergetics in response to caloric restriction alone or in combination with exercise in 36 young (36.8 ± 1.0 y), overweight (body mass index, 27.8 ± 0.7 kg/m^2^) individuals randomized into one of three groups for a 6-mo intervention: Control, 100% of energy requirements; CR, 25% caloric restriction; and CREX, caloric restriction with exercise (CREX), 12.5% CR + 12.5% increased energy expenditure (EE). In the controls, 24-h EE was unchanged, but in CR and CREX it was significantly reduced from baseline even after adjustment for the loss of metabolic mass (CR, −135 ± 42 kcal/d, *p* = 0.002 and CREX, −117 ± 52 kcal/d, *p* = 0.008). Participants in the CR and CREX groups had increased expression of genes encoding proteins involved in mitochondrial function such as PPARGC1A, TFAM, eNOS, SIRT1, and PARL (all, *p* < 0.05). In parallel, mitochondrial DNA content increased by 35% ± 5% in the CR group (*p* = 0.005) and 21% ± 4% in the CREX group (*p* < 0.004), with no change in the control group (2% ± 2%). However, the activity of key mitochondrial enzymes of the TCA (tricarboxylic acid) cycle (citrate synthase), beta-oxidation (beta-hydroxyacyl-CoA dehydrogenase), and electron transport chain (cytochrome C oxidase II) was unchanged. DNA damage was reduced from baseline in the CR (−0.56 ± 0.11 arbitrary units, *p* = 0.003) and CREX (−0.45 ± 0.12 arbitrary units, *p* = 0.011), but not in the controls. In primary cultures of human myotubes, a nitric oxide donor (mimicking eNOS signaling) induced mitochondrial biogenesis but failed to induce SIRT1 protein expression, suggesting that additional factors may regulate SIRT1 content during CR.

**Conclusions:**

The observed increase in muscle mitochondrial DNA in association with a decrease in whole body oxygen consumption and DNA damage suggests that caloric restriction improves mitochondrial function in young non-obese adults.

## Abstract 2

Anthony Civitarese and colleagues observed an increase in mitochondrial DNA in muscle and a decrease in whole body oxygen consumption in healthy adults who underwent caloric restriction.

## Editors' Summary

**Background.:**

Life expectancy (the average life span) greatly increased during the 20th century in most countries, largely due to improved hygiene, nutrition, and health care. One possible approach to further increase human life span is “caloric restriction.” A calorie-restricted diet provides all the nutrients necessary for a healthy life but minimizes the energy (calories) supplied in the diet. This type of diet increases the life span of mice and delays the onset of age-related chronic diseases such as heart disease and stroke. There are also hints that people who eat a calorie-restricted diet might live longer than those who overeat. People living in Okinawa, Japan, have a lower energy intake than the rest of the Japanese population and an extremely long life span. In addition, calorie-restricted diets beneficially affect several biomarkers of aging, including decreased insulin sensitivity (a precursor to diabetes). But how might caloric restriction slow aging? A major factor in the age-related decline of bodily functions is the accumulation of “oxidative damage” in the body's proteins, fats, and DNA. Oxidants—in particular, chemicals called “free radicals”—are produced when food is converted to energy by cellular structures called mitochondria. One theory for how caloric restriction slows aging is that it lowers free-radical production by inducing the formation of efficient mitochondria.

**Why Was This Study Done?:**

Despite hints that caloric restriction might have similar effects in people as in rodents, there have been few well-controlled studies on the effect of good quality calorie-reduced diets in healthy people. It is also unknown whether an energy deficit produced by increasing physical activity while eating the same amount of food has the same effects as caloric restriction. Finally, it is unclear how caloric restriction alters mitochondrial function. The Comprehensive Assessment of Long-term Effects of Reducing Intake of Energy (CALERIE) organization is investigating the effect of caloric restriction interventions on physiology, body composition, and risk factors for age-related diseases. In this study, the researchers have tested the hypothesis that short-term caloric deficit (with or without exercise) increases the efficiency of mitochondria in human muscle.

**What Did the Researchers Do and Find?:**

The researchers enrolled 36 healthy overweight but non-obese young people into their study. One-third of them received 100% of their energy requirements in their diet; the caloric restriction (CR) group had their calorie intake reduced by 25%; and the caloric restriction plus exercise (CREX) group had their calorie intake reduced by 12.5% and their energy expenditure increased by 12.5%. The researchers found that a 25% caloric deficit for six months, achieved by diet alone or by diet plus exercise, decreased 24-hour whole body energy expenditure (i.e., overall calories burned for body function), which suggests improved mitochondrial function. Their analysis of genes involved in mitochondria formation indicated that CR and CREX both increased the number of mitochondria in skeletal muscle. Both interventions also reduced the amount of DNA damage—a marker of oxidative stress—in the participants' muscles.

**What Do These Findings Mean?:**

These results indicate that a short-term caloric deficit, whether achieved by diet or by diet plus exercise, induces the formation of “efficient mitochondria” in people just as in rodents. The induction of these efficient mitochondria in turn reduces oxidative damage in skeletal muscles. Consequently, this adaptive response to caloric restriction might have the potential to slow aging and increase longevity in humans as in other animals. However, this six-month study obviously provides no direct evidence for this, and, by analogy with studies in rodents, an increase in longevity might require lifelong caloric restriction. The results here suggest that even short-term caloric restriction can produce beneficial physiological changes, but more research is necessary before it becomes clear whether caloric restriction should be recommended to healthy individuals.

**Additional Information.:**

Please access these Web sites via the online version of this summary at http://dx.doi.org/10.1371/journal.pmed.0040076.

- The CALERIE (Comprehensive Assessment of Long-Term Effects of Reducing Intake of Energy) Web site contains information on the study and how to participate
- American Federation for Aging Research includes information on aging with pages on the biology of aging and on caloric restriction
- The Okinawa Centenarian Study is a population-based study on long-lived elderly people in Okinawa, Japan
- US Government information on nutrition
- MedlinePlus encyclopedia pages on diet and calories
- The Calorie Restriction Society, a nonprofit organization that provides information on life span and caloric restriction
- Wikipedia pages on calorie restriction and on mitochondria (note: Wikipedia is an online encyclopedia that anyone can edit)

## Introduction

Caloric restriction delays the rate of aging in many species such as yeast, worms, flies, and mice [1]. One theory is that caloric restriction reduces oxidative damage to proteins, lipids, and DNA, although the underlying mechanisms of this process are unclear. Cellular nutrient sensing systems seem to mediate many of the metabolic responses to caloric restriction, including the regulation of free radical production and oxidative stress. Mitochondria are the major consumers of cellular oxygen (~85%) [2] and the predominant production site of free radicals, a by-product of oxidative phosphorylation [3]. Studies in mammals have shown that caloric restriction reduces the generation of free radicals by mitochondria, in parallel to reductions in mitochondrial proton leak [4,5] and whole-body EE [4] (defined as oxidation or combustion of food and/or energy stores to meet the body's energy requirements). Paradoxically, caloric restriction induces mitochondrial proliferation in rodents [6,7], and either lowers [8] or does not affect mitochondrial oxygen consumption [6]. Taken together, these data suggest that caloric restriction improves whole body energy efficiency by inducing the biogenesis of mitochondria that utilize less oxygen and produce less reactive oxygen species (ROS).

Most research on the transcriptional regulation of mitochondrial number and function focuses on downstream transcription factors such as nuclear respiratory factors (NRF) 1 and 2, and transcription factor A, mitochondrial (TFAM). These transcription factors coordinate the transcription of both the nuclear and the mitochondrial genes involved in mitochondrial bioenergetics [9,10]. The *peroxisome proliferator-activated receptor gamma coactivator 1α* (*PPARGC1A)* lies upstream of *NRF1* and *TFAM,* and the protein serves as a nutrient sensing system that increases mitochondrial biogenesis and shifts substrate utilization toward fat and away from carbohydrate [11]. A molecular regulator of *PPARGC1A* is Sir2, a nicotinamide adenine dinucleotide (NAD)-dependent deacetylase that prolongs life span in yeast when overexpressed [12]. The mechanism through which Sir2 extends life span is unclear, although data show that Sir2-mediated deacetylation of modified lysine residues on histones, transcription factors, and other nuclear substrates is involved [11,13,14]. The mammalian ortholog of Sir2, known as sirtuin 1 (SIRT1), deacetylates and activates *PPARGC1A* [11,14], suggesting a direct link between SIRT1 and mitochondrial bioenergetics. Currently, there no data in human skeletal muscle on how caloric restriction may affect the expression of *PPARGC1A* and *SIRT1,* as well as mitochondrial number and function.

Research strongly supports the health benefits of exercise in humans of all ages. Increased exercise in the absence of other behavioral changes prevents the onset of many chronic diseases [15]. In addition, the combination of weight loss and exercise in overweight individuals increases insulin sensitivity [16], *PPARGC1A* gene expression [17], and mitochondrial function [16]. However, to our knowledge no study has addressed the interactive effects of diet and exercise on skeletal muscle mitochondrial function in non-obese humans. The aims of this study was to test the hypothesis that short-term caloric deficit (caloric restriction with or without exercise) would coordinately up-regulate the expression of genes involved in mitochondrial biogenesis in skeletal muscle resulting in increased mitochondrial content, improved whole body energy efficiency, and decreased DNA fragmentation in non-obese humans.

## Methods

### Participants

Participants were recruited as previously described [18] and gave their written informed consent to provide biopsy samples for muscle molecular analysis. However, the specific muscle bioenergetic tests conducted in this study were not prespecified (see Protocol S1). Briefly, healthy, male (25–50 y) and female (25–45 y), overweight participants (body mass index, 25 to <30 kg/m^2^) were enrolled in the study (trial registration: http://www.ClinicalTrials.gov; identifier: NCT00099151). The baseline period was conducted over 5 wk to carefully determine individual energy requirements by two 2-wk measures of doubly labeled water during weight maintenance [18]. The participants then attended a 5-d inpatient stay during which metabolic tests were conducted and the 5-d inpatient stay was repeated after 6 mo. Individuals (*n* = 36) were randomized into one of three groups for 6 mo: Control, weight maintenance diet; CR, 25% calorie restriction of baseline energy requirements; and caloric restriction with exercise (CREX), 12.5% CR + 12.5% increase in EE by structured exercise as previously described [18]. All diets were based on the American Heart Association recommendations (≤30% fat). CREX participants increased EE by 12.5% above baseline energy requirements by undergoing structured exercise (walking, running, stationary cycling) 5 d/wk with three sessions under direct supervision and two with heart rate monitoring. EE was measured in a respiratory chamber [18]. The Internal Review Board of the Pennington Biomedical Research Center and the data safety monitoring board of CALERIE approved the protocol, and participants provided written informed consent.

### Biochemical Analysis

Body fat was measured by dual energy x-ray absorptiometry (QDA 4500A, Hologics, http://www.hologic.com). Fasting serum insulin and tri-iodothyronine (T_3_) levels were measured using immunoassays (DPC 2000, Diagnostic Products Corporation, http://www.dpc.com). Plasma glucose was analyzed using a glucose oxidase electrode (Synchron CX7, Beckman, http://www.beckmancoulter.com), whereas adiponectin was determined using nonradioactive ELISA (Linco, http://www.lincoresearch.com).

### mRNA Quantification of Gene Expression

Vastus lateralis biopsy samples (~150 mg) were taken after local anesthesia using the Bergstrom technique [19]. Fat and connective tissue was removed from the biopsy sample and muscle was snap frozen in liquid nitrogen and stored at −80°C until completion of the study. RNA was isolated from approximately 30 mg of tissue using the acid phenol method and purified with RNeasy columns (Qiagen, http://www.qiagen.com). DNA was extracted from the same tissue samples, after degradation of protein and RNA with Trizol, by phenol-chloroform extraction and ethanol precipitation according to the manufacturer's procedure (Invitrogen, http://www.invitrogen.com). Primers and probes (Table S1) were designed using Beacon Designer V2.1 (Bio-Rad, http://www.bio-rad.com). qRT (quantitative real-time)-PCR reactions were performed as previously described [20]. All expression data were normalized by dividing the target gene by RPLPO or *peptidylprolyl isomerase B (PPIB)* mRNA (which encodes cyclophilin B).

### Real-Time PCR for Mitochondrial DNA

Relative amounts of nuclear DNA and mitochondrial DNA (mtDNA) were determined by qRT-PCR as previously described [21]. The ratio of mtDNA to nuclear DNA reflects the tissue concentration of mitochondria per cell.

### Skeletal Muscle Mitochondrial Enzyme Activities

Citrate synthase, beta-hydroxyacyl-CoA dehydrogenase and cytochrome C oxidase II activities were determined spectrophotometrically in muscle homogenates using previously described methods [22]. Briefly, about 50 mg of skeletal muscle was weighed, diluted 20-fold in extraction buffer and then homogenized (0.1 M KH_2_PO_4_/Na_2_PHO_4_, 2 mM EDTA [pH = 7.2]). Citrate synthase activity was measured at 37 °C in 0.1 M Tris-HCl (pH 8.3) assay buffer containing 0.12 mM 5,5′-dithio-bis 2-nitrobenzoic acid and 0.6 mM oxaloacetate. After an initial 2-min absorbance reading at 412 nm, the reaction was initiated by adding 3.0 mM acetyl-CoA, and the change in absorbance was measured every 10 s for 7 min. Beta-hydroxyacyl-CoA dehydrogenase activity was measured at 37 °C in assay buffer containing 0.1 M triethanolamine-HCl, 5 mM EDTA, and 0.45 mM NADH (pH 7.0). After an initial 1-min absorbance reading at 340 nm, the reaction was initiated by adding 0.1 mM acetoacetyl-CoA, and the change in absorbance was measured every 10 s for 5 min. Cytochrome C oxidase II was measured at 25 °C in 0.03 M potassium phosphate buffer containing reduced cytochrome C (2 mg/ml) and 4 mM sodium hydrosulfite. The reaction was initiated by the addition of sample and the change of absorbance was measured every 10 s for 5 min at 550 nm and values corrected for total protein (mg/ml).

### Antioxidant Enzyme Activity

Cell lysates were prepared by sonication in cold 20 mM HEPES buffer (pH 7.2), containing 1 mM EDTA, 210 mM mannitol, and 70 mM sucrose and centrifuged at 1,500 *g* for 5 min at 4 °C. Total superoxide dismutase (SOD) activity (Cu/Zn-, Mn-, and Fe-SOD) was quantitated by measuring the dismutation of superoxide radicals generated by xanthine oxidase. Enzyme activity was determined by an indirect assay according to the commercially available Cayman Chemicals assay kit (http://www.caymanchem.com).

### Mitochondrial Membrane Potential and Mitochondrial Mass

Measurement of mitochondrial membrane potential tetramethylrhodamine ethyl ester (TMRE; Molecular Probes, http://probes.invitrogen.com) was performed as previously described [22]. For quantification of mitochondrial mass we used Mitotracker Green probe (Molecular Probes). Mitotracker Green probe preferentially accumulates in mitochondria regardless of the mitochondrial membrane potential and provides an accurate assessment of mitochondrial mass. Cells were washed with PBS and incubated at 37 °C for 30 min with 100 nM MitoTracker Green FM (Molecular Probes). Cells were harvested using trypsin/EDTA and resuspended in PBS. Fluorescence intensity was detected with excitation and emission wavelengths of 490 and 516 nm, respectively, and values corrected for total protein (mg/ml).

### Skeletal Muscle Cell Culture

Vastus lateralis muscle biopsies (~100 mg) were obtained from five healthy, normal-weight, young, sedentary individuals (body mass index = 24.1 ± 0.5 kg/m^2^; body fat = 15% ± 1%; fasting glucose concentration = 88 ± 2 mg/dl; fasting plasma insulin = 4.4 ± 0.9 μU/ml). Satellite cells were isolated as previously described [23]. Cells were seeded into 12-well (RNA/DNA) and six-well plates (protein) at a density of 20 × 10^3^ cells/cm^2^. Cells were grown at 37 °C in a humidified atmosphere of 5% CO_2_. Two pairs of Stealth-small interfering (si) RNAs were chemically synthesized (Invitrogen), annealed, and transfected (200 pmol/ml) into 50%–70% confluent primary human myocytes using GeneSilencer siRNA transfection reagents (Gene Therapy Systems, http://www.genetherapysystems.com). The sequences of the sense siRNAs are as follows; AdipoR1, 5′-AAACAGCACGAAACCAAGCAGAUGG-3′; AdipoR2, 5′-AUGUCCCACUGGGAGACUAUAAUGG-3′. Cells transfected with nonfunctional-jumbled siRNA (mock) (Ambion, http://www.ambion.com) were used as negative controls (unpublished data). Differentiation of myoblasts into myotubes was initiated as previously described [23]. At 72 h after induction of differentiation, the medium was changed and treated with either mammalian globular adiponectin for 48 h (R&D Systems, Minneapolis, MN, USA) or with a nitric oxide donor— 50 μM of 2,2′-(hydroxynitrosohydrazono)bis-ethanimine (DETA-NO) (Sigma, http://www.sigmaaldrich.com)—once a day for 4 d [7,24].

### Statistical Methods

Data are expressed as means ± standard errors of the mean. Data analysis was performed using SAS v. 9.1 (http://www.sas.com). Changes from baseline to month 6 were analyzed by analysis of covariance with treatment and time interactions and baseline value included as a covariate. In cell culture experiments, t-tests were used to determine treatment effect. Pearson or Spearman correlations were used where appropriate. Data were considered significant if *p* < 0.05.

## Results

### Caloric Restriction Reduces Whole-Body Energy Expenditure

Physical and metabolic characteristics of the participants are given in Table 1 and described elsewhere [18]. Body weight was significantly reduced in CR (−10.4% ± 0.9% kg, *p* < 0.001) and CREX (−10.0% ± 0.8% kg, *p* < 0.001) groups relative to controls (−1.0% ± 1.1% kg) with significant losses in fat mass (CR, −24% ± 3% kg and CREX, −25% ± 3% kg; both *p* < 0.001) and fat-free mass (FFM) (CR, −5% ± 1% kg and CREX −3% ± 1% kg, both *p* < 0.001) but no difference between the two intervention groups [18]. Absolute 24-h EE was significantly reduced from baseline to month 6 in CR and CREX (*p* < 0.001, Table 1). This decrease in 24-h EE remained significant after adjustment for metabolic body size changes in FFM (CR, −135 ± 42 kcal/d, *p* = 0.002 and CREX, −117 ± 52 kcal/d, *p* = 0.008) [18]. Simultaneously, after 6-mo of intervention, plasma triiodothyronine (T_3_) concentration decreased in CR (−8.9 ng/ml, *p* < 0.001) and CREX groups (−4.5 ng/ml, *p* = 0.01), suggesting that T_3_ may contribute to lower sedentary energy expenditure [25]. DNA damage was reduced from baseline in the CR (−0.56 ± 0.11 arbitrary units, *p* = 0.003) and CREX participants (−0.45 ± 0.12 arbitrary units, *p* = 0.011) but not in the controls [18]. In addition, the activity of superoxide dismutase (SOD), a free radical scavenging enzyme that catalyzes the breakdown of ROS to hydrogen peroxide and molecular oxygen, tended to decrease in the skeletal muscle of the CR group (−22% ± 9%, *p* = 0.15).

**Table 1:**
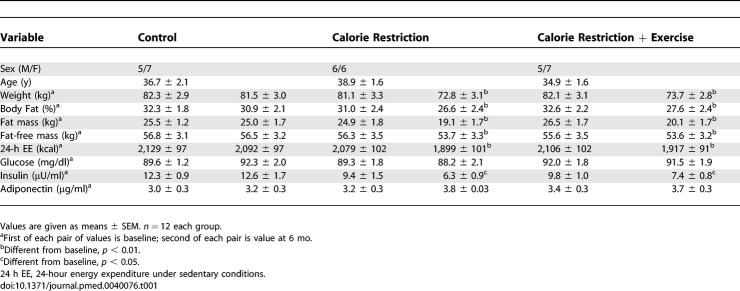
Metabolic Characteristics of Subjects Completing the Study (*n* = 36)

Fasting glucose was not changed in any of the groups, whereas fasting insulin was significantly reduced from baseline in the CR and CREX groups (Table 1), consistent with an expected improvement in insulin sensitivity [26–28]. The potential importance of adiponectin as a mediator of the effects of caloric restriction is underscored by studies in rodents [29] and humans [30] demonstrating that caloric restriction improves insulin sensitivity in parallel with an increase in circulating adiponectin. In our study, total plasma adiponectin tended to increase in both intervention groups by 17% ± 1% in CR and 7% ± 1% in CREX (Table 1), possibly due to the small sample size and/or differential regulation of the multimeric forms of adiponectin.

### Caloric Restriction Increases Mitochondrial Content in Skeletal Muscle

**Figure 1:**
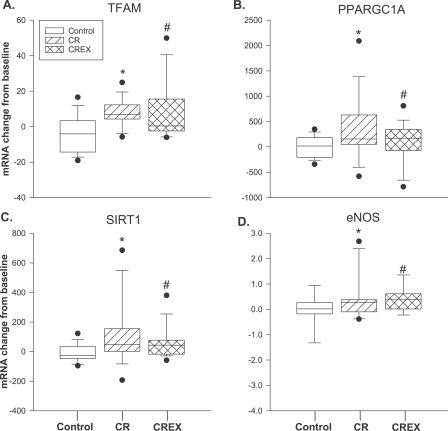
Changes in Skeletal Muscle Gene Expression for Key Mitochondrial Proteins (A) TFAM: ^*^CR, *p* = 0.001; ^#^CREX, *p* = 0.014. (B) PPARGC1A: ^*^CR, *p* = 0.004; ^#^CREX, *p* = 0.002. (C) SIRT1: ^*^CR, *p* = 0.016; ^#^CREX, *p* = 0.023. (D) eNOS: ^*^CR, *p* = 0.002; ^#^CREX, *p* = 0.039. Graphs show six-month changes in enzyme expression in response to each intervention. The y-axis represents the relative gene expression change from baseline for each study group. Each box plot shows the distribution of expression levels from 25th to 75th percentile, and the lines inside the boxes denote the medians. The whiskers denote the interval between the 10th and 90th percentiles. The filled circles mark the data points outside the 10th and 90th percentiles. Molecular analysis was performed in 11 of 12 volunteers per group from whom there was sufficient pre- and postintervention sample for this determination. Changes from baseline to month 6 were analyzed by analysis of variance with baseline values included as covariates.

**Figure 2:**
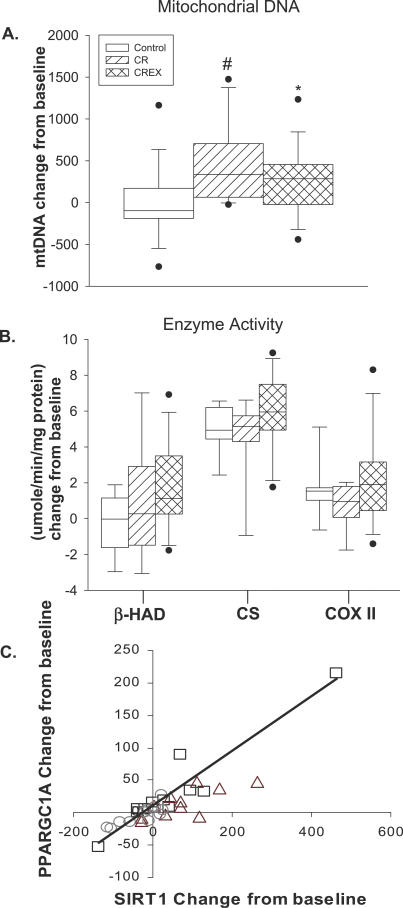
The Effects of Caloric Restriction on Mitochondrial Bionenergetics (A and B) Each box plot shows the distribution of expression levels from 25th to 75th percentile and the lines inside the boxes denote the medians. The whiskers denote the interval between the 10th and 90th percentiles. The filled circles mark the data points outside the 10th and 90th percentiles. (A) Caloric deficit–induced mitochondrial biogenesis in the CR group (35% ± 5%, ^*^ *p* = 0.005) and the CREX group (21% ± 4%, ^#^ *p* < 0.004), with no change in the control group (2% ± 2%). The y-axis represents the relative change from baseline in mtDNA for each study group. (B) Analysis of mitochondrial enzyme activity; β-HAD (β-oxidation); CS (TCA cycle), and COX (electron transport chain). The y-axis represents the relative change from baseline in mitochondrial enzyme activity for each study group. (C) Linear correlation between the change from baseline in *SIRT1* and *PPARGC1A* mRNAs from baseline in control (○), *r* = 0.83, *p* < 0.05; CR (□), *r* = 0.95, *p* < 0.01; and CREX participants (▵), *r* = 0.76, *p* < 0.05). The linear correlation between the change in *SIRT1* mRNA and *PPARGC1A* mRNA from baseline in the CR group (□) remained significant after exclusion of the outlier (*r* = 0.81, *p* < 0.01). Changes from baseline to month 6 were analyzed by analysis of variance with baseline values included as covariates.

Six months of caloric restriction caused an increase in the expression levels of *TFAM* (the principal transcription factor involved in regulating mtDNA transcription) and *PPARGC1A* (Figure 1A and 1B), suggesting an induction of mitochondrial biogenesis. Consistently, there was a significant induction in mtDNA content (a marker for mitochondrial mass [31,32]) in CR (35% ± 5%; *p* = 0.005) and CREX (21% ± 4%; *p* = 0.004) groups with no change in the control group (2% ± 2%) (Figure 2A). However, in the three groups, we did not observe any change in citrate synthase protein content, another marker of mitochondrial mass (unpublished data). The activity of beta-hydroxyacyl-CoA dehydrogenase (a measure of β-oxidation), citrate synthase (a measure of TCA cycle activity), and cytochrome C oxidase II (a measure of electron transport chain activity) did not change in response to CR or CREX (Figure 2B).

We next examined the skeletal muscle expression of genes involved in oxidative transcription, mitochondrial oxidation, and oxidative stress (see Table S1 for complete listing of genes). *SIRT1* mRNA was increased in the CR (113% ± 24%; *p* = 0.016) and CREX groups (61% ± 8%; *p* = 0.023) (Figure 1C). Baseline mRNA expression of *PPARGC1A* and *SIRT1* were highly correlated in all groups (control, *r* = 0.81, *p* < 0.001; CR, *r* = 0.86, *p* < 0.001; and CREX, *r* = 0.85, *p* < 0.001). Furthermore, the changes from baseline to 6-mo in *PPARGC1A* and *SIRT1* were strongly correlated in all participants and in each group separately (Figure 2C). *Endothelial nitric oxide synthase (eNOS)* gene expression was increased by 67% ± 19% (*p* = 0.002) and 66% ± 16% (*p* = 0.039) in the CR and CREX groups, respectively (Figure 1D). The change in *eNOS* mRNA from baseline was highly correlated with the change in the expression of *PPARGC1A* and *SIRT1* in the CR group (*r* = 0.86, *p* < 0.001 and *r* = 0.80, *p* < 0.001, respectively; unpublished data), suggesting synergistic regulation among PPARGC1A, SIRT1, and eNOS. Furthermore, *presenilin-associated, rhomboid-like protein (PARL)* and AMP-activated protein kinase alpha 2 (AMPK-α2) mRNA increased relative to baseline in the CR group only (*PARL,* 25 ± 3 versus 54 ± 11, *p* = 0.008; AMPK-α2, 387 ± 26 versus 521 ± 59 mRNA/ribosomal protein large, PO (RPLPO) mRNA, *p* = 0.03).

### Nitric Oxide and Mitochondrial Biogenesis in Primary Human Muscle Cell Culture

**Figure 3:**
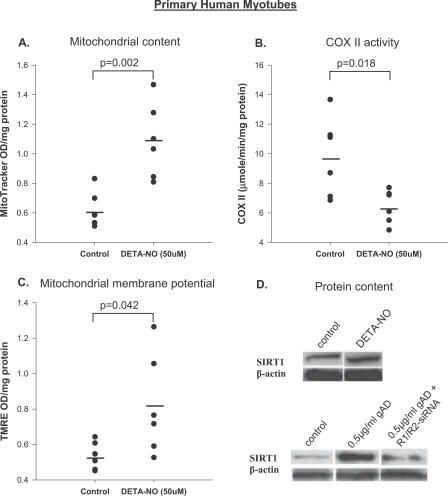
Statistical Dot Plots Showing the Effects of DETA-NO and Adiponectin Treatment on Mitochondrial Content and SIRT1 Protein in Primary Human Myotubes (A–C) Effects of 96 h of 50 μM DETA-NO treatment on mitochondrial content (using MitoTracker Green, *p* = 0.002) (A), electron transport chain activity (COX, *p* = 0.018) (B), and mitochondrial membrane potential (TMRE, *n* = 6, *p* = 0.042) (C). Treatment effect was determined using independent sample t-test. OD, optical density. (D) Effects of 50 μM DETA-NO on SIRT1 and β-actin protein (top blots); effects of 0.5 μg/ml of globular adiponectin (gAD) and adiponectin receptor R1- and R2-siRNA on SIRT1 and β-actin protein expression (bottom blots). Immunoblotting was undertaken in three participants and data are shown as a representative blot. Means are denoted by the solid black bars.

To determine whether nitric oxide (NO) (mimicking eNOS activity) was associated with mitochondrial biogenesis in human skeletal muscle, we treated primary human myotubes (see Methods for clinical characteristics of the donors) with the NO donor DETA-NO. A 4-d treatment with 50 μM of DETA-NO increased the mRNA expression of *TFAM* (0.62 ± 0.1 versus 1.3 ± 0.1, *p* < 0.001) and induced mitochondrial biogenesis (indicated by MitotrackerGreen, Figure 3A). DETA-NO treatment decreased cytochrome C oxidase II activity (structural component of complex IV; Figure 3B) and increased mitochondrial membrane potential using TMRE (Figure 3C).

### Adiponectin Signaling Increases SIRT1 Protein

Examination of SIRT1 mRNA (unpublished data) and protein content (Figure 3D, top) in DETA-NO–treated cells demonstrated no difference relative to untreated cells. However, a 2-d treatment of human myotubes with 0.5 μg/ml globular adiponectin increased SIRT1 protein (Figure 3D, bottom) but had no effect on *SIRT1* mRNA (unpublished data), indicating post-transcriptional regulation. Knocking down adiponectin receptor (-R1 and -R2 isoforms) gene expression by siRNA blunted the adiponectin-induced SIRT1 protein increase in primary human myotubes (Figure 3D, bottom).

## Discussion

The current study investigated the impact of caloric restriction on muscle mitochondrial function in healthy overweight but non-obese humans. The study provides, to our knowledge, the first evidence that a 25% caloric deficit either by caloric restriction alone or by a combination of caloric restriction and exercise decreased 24 h EE and improved mitochondrial function. Molecular analysis revealed that CR and CREX increased the expression of genes involved in nutrient sensing and mitochondrial biogenesis as well as increasing mitochondrial mass without a change in the activity of key mitochondrial enzymes involved in the Krebs cycle, β-oxidation, and electron transport chain activity. However, caloric restriction decreased markers of oxidative stress. Our results suggest that caloric restriction induces biogenesis of “efficient” mitochondria as an adaptive mechanism, which in turn lowers oxidative stress.

### Caloric Restriction Improves Whole-Body Metabolic Efficiency and Lowers Markers of Oxidative Stress

One proposed explanation for the beneficial effects of caloric restriction is reduced oxygen consumption, which in turn lowers mitochondrial ROS production [33]. However, published studies have had conflicting results. In healthy primates, ten to 11 years of caloric restriction lowers resting EE, beyond the decrease attributed to losses in FFM and fat mass [34,35]. In rodents, reports have been more divisive with decreased, no change, or increased EE in response to caloric restriction [7,36–39]. In humans we now show that short-term caloric restriction with or without exercise reduces fat mass, FFM, and absolute 24-h EE. However, after correcting for changes in FFM and fat mass, 24-h EE was still lowered by CR or CREX, suggesting that energy deficit rather than caloric restriction per se caused the increase in energy efficiency. These results are consistent with studies in obese and lean individuals in which a 10%–15% reduction in energy requirement was reported for weight maintenance after adjustment for FFM [40].

Improvement in whole-body oxygen consumption may therefore be expected to lower ROS production, a normal byproduct of oxidative phosphorylation. ROS are formed by single electron transfer to oxygen generating super oxide radicals (O_2_^•−^), hydrogen peroxide (H_2_O_2_), and hydroxyl radicals (OH^•^) [3,41]. ROS react with DNA, protein, and lipids, leading to oxidative damage. Previous studies have shown that caloric restriction decreases proton leaks and oxidative stress damage [33,42]. Low mitochondrial content seems to contribute to increased ROS production [41]. When mitochondrial mass is reduced, mitochondria have increased “workload,” leading to higher membrane potential and increased ROS production [3,6,7,41]. Increased mitochondrial mass in response to caloric restriction may therefore be an important adaptive mechanism to reduce oxidative stress without a necessary decrease in cellular respiration [6,8]. In our study, DNA damage was reduced from baseline in both the CR and CREX participants with a tendency toward lower SOD activity. These data are consistent with the hypothesis that caloric restriction induces the proliferation of efficient mitochondria and reduces oxidative damage to mtDNA and cellular organelles.

### Caloric Restriction Improves Mitochondrial Function

To investigate the molecular mechanism(s) by which caloric restriction lowered oxygen consumption per unit of FFM, we determined expression of genes involved in mitochondrial biogenesis including *PPARGC1A, PPARGC1B, NRF1,* and *TFAM* in skeletal muscle—a metabolically active organ [43] reported to be involved in the aging process [44] and oxidative stress [6]. Six months of caloric restriction caused an increase in the expression levels of *TFAM* and *PPARGC1A* and induced mitochondrial biogenesis. Surprisingly, citrate synthase protein was unchanged in all groups. Several investigators have shown a dissociation between mitochondrial mass and in vitro mitochondrial oxidative capacity after caloric restriction [45–48]. In addition, mice treated with the caloric restriction “mimetic” compound resveratrol display elevated hepatic mitochondrial content that is independent of citrate synthase activity [49]. In line with these results, the activity of citrate synthase, beta-hydroxyacyl-CoA dehydrogenase and cytochrome C oxidase II activity did not change in response to interventions. Our results are in agreement with previous reports indicating oxidative enzyme activity does not improve after weight loss in obese humans [50,51]. Taken together, our results suggest: (1) caloric restriction induces the proliferation of mitochondria with “efficient” electron transport systems coupled to lower whole-body oxygen consumption, and (2) mtDNA or other nonenzymatic markers of mitochondrial mass (i.e., mitochondrial structural proteins such as cardiolipin [52]) may be better indicators of mitochondrial content than enzyme activities under caloric restriction conditions in humans. In support of this latter hypothesis, *PARL* gene expression was higher in the CR group at the end of the intervention. PARL is a mitochondrial-specific rhomboid protease involved in the regulation of inner mitochondrial membrane function, structure [7], and apoptosis [53]. A similar induction in mitochondrial fusion gene expression (mitofusin 1 and 2) has been reported after caloric restriction in rodents [7].

### Caloric Restriction Increases Expression of Genes Involved in Mitochondrial Biogenesis and Nutrient Sensing

In rodents, the expression of SIRT1 is increased in response to prolonged caloric restriction in adipose, liver, kidney, and brain tissue [7,54]. SIRT1 is known to bind, deacetylate, and activate PPARGC1A, thus promoting mitochondrial biogenesis [11]. Such results raise the possibility that SIRT1 may improve mitochondrial function and lower oxidative damage in humans. In support of this concept, *SIRT1* mRNA was increased in the CR and CREX groups in proportion to the increase in *PPARGC1A* mRNA, consistent with the potential role of SIRT1 as a direct regulator of *PPARGC1A* activity [11,14]. We observed a smaller induction in mitochondrial biogenesis and *SIRT1* mRNA in the CREX relative to CR group. The disparity in the molecular adaptation between CR and CREX groups may relate to SIRT1 activity and the ratio of NAD^+^/NADH, which oscillates depending on the rate of glycolysis [55] and during submaximal exercise [56]. This might be expected to change SIRT1 activity and gene transcription. Conversely, aerobic exercise can independently improve muscle metabolic efficiency [57,58] by a contraction-stimulated increase in mitochondrial biogenesis [59] and the expression of genes encoding proteins involved in mitochondrial function including *PPARGC1A, PPAR-δ,* and *PGC-1-related coactivator* [17,60].

In rodents, knockout of the *eNOS* gene abolishes the caloric restriction-induced expression of *PPARGC1A* and *TFAM* mRNA and mitochondrial protein content [7]. Consistently, *eNOS* gene expression was increased in both intervention groups, suggesting an important role of eNOS in mitochondrial biogenesis in human muscle. In various cell systems, NO treatment induces mitochondrial biogenesis [61] via a *PPARGC1A*-dependent mechanism [7,24]. In our study, NO treatment of primary cultures of human muscle induced mitochondrial biogenesis. These data are consistent with studies in human U937 cells and rodent L6 myoblast [7,24]. This pleiotrophic effect occurs by NO diffusing from the mitochondria to the cytosol in response to a high mitochondrial membrane potential [62]. NO is a potent inhibitor of oxygen consumption and oxidative phosphorylation by inhibition of complex IV [63,64]. Consistently, we observed reduced cytochrome C oxidase II activity and increased mitochondrial membrane potential in primary human myotubes treated with DETA-NO. Both NO and high mitochondrial membrane potential are potent inducers of ROS production [3,65]. Taken together, these data suggest that NO can induce mitochondrial biogenesis in human skeletal muscle, but would also promote the production of ROS vis-à-vis elevated mitochondrial membrane potential and reduced electron transport chain activity.

### Adiponectin Induces Expression of SIRT1 Protein

In murine white adipocytes NO treatment increases SIRT1 protein [7], suggesting eNOS may also be involved in regulating SIRT1 in skeletal muscle. In this study, NO treatment did not change SIRT1 protein content in primary human myotubes. A possible discrepancy between the study by Nisoli et al. [7] and ours may relate to tissue- or pathway-specific regulation of SIRT1 [7,11]. However, our results indicate that NO can induce mitochondrial biogenesis in human skeletal muscle without increasing SIRT1 protein expression. This suggests that additional factors may be involved in improving mitochondrial function and regulating SIRT1 activity in response to caloric restriction in human muscle. Interestingly, we observed an increase in *AMPK-α2* mRNA in the CR group, suggesting increased AMPK activity. Previous reports from our laboratory demonstrated that increased signaling through an adiponectin-AMPK interaction stimulates PPARGC1A protein expression and mitochondrial biogenesis, and lowers ROS production in human skeletal muscle [32]. Consistently, adiponectin-mediated signaling increased SIRT1 protein in primary human myotubes. These data indicate that adiponectin signaling may be involved in the regulation of SIRT1-PPARGC1A protein expression. We hypothesize that some of the metabolic benefits induced by caloric restriction may result from improved secretion of adipokines such as adiponectin.

A widely accepted view is that long-term caloric restriction improves mitochondrial oxidative phosphorylation [6], therefore lowers ROS production and oxidative damage. We show, to our knowledge for the first time, that in overweight nonobese humans, short-term caloric restriction lowers whole-body energy expenditure (metabolic adaptation), in parallel with an induction in mitochondrial biogenesis, *PPARGC1A* and *SIRT1* mRNA, and a decrease in DNA damage. We therefore propose that caloric restriction induces biogenesis of “efficient” mitochondria in human skeletal muscle as an adaptive mechanism, which in turn lowers oxidative stress.

## Supporting Information

Protocol S1 CALERIE Study Institutional Review Board–Approved Clinical Trial Protocol (250 KB PDF)

Table S1 Oligonucleotide Sequences for Primer/Probe Sets Used in TaqMan Analysis (50 KB DOC)

## Abbreviations

CR: 25% caloric restriction group
CREX: caloric restriction group with 12.5% caloric restriction + 12.5% increased energy expenditure
DETA-NO: 2,2′-(hydroxynitrosohydrazono)bis-ethanimine
EE: energy expenditure
FFM: fat-free mass
mtDNA: mitochondrial DNA
NO: nitric oxide
qRT-PCR: quantitative real-time PCR
ROS: reactive oxygen species
siRNA: small interfering RNA
TMRE: tetramethylrhodamine ethyl ester.
