# Supplementary material for: Calorie Restriction Increases Muscle Mitochondrial Biogenesis in Healthy Humans
Source: PLoS Med. 2007 Mar 6;4(3):e76. doi: 10.1371/journal.pmed.0040076 (PMC1808482; doi:10.1371/journal.pmed.0040076)
Supplement: Supplementary file 1 [file pmed.0040076.sd001.pdf]

METABOLIC ADAPTATIONS TO SIX-MONTH CALORIC RESTRICTION

Eric Ravussin, Ph.D. (P.I.) and Donald Williamson, Ph.D.

Frank Greenway, M.D. and Steve Smith, M.D., (Medical Investigators),

And

Claude Bouchard, Ph.D., James DeLany, Ph.D., Andrew Deutsch, Ph.D., Paula J. Geiselman, Ph.D., D. Enette Larson-Meyer, R.D., Ph.D., Michael Lefevre, Ph.D., Marlene Most-Windhauser, R.D., Ph.D., Leonie Heilbronn, Ph.D. (Co-Investigators)

BACKGROUND AND SIGNIFICANCE

It was reported 60 years ago that caloric restriction (CR) extended lifespan and retarded age-related chronic diseases. This was first described in the 1930s by McCay et al [1]. Since then, similar observations have been made in a variety of animal species including rats, mice, fish, flies, worms and yeast [2,3]. Recent observations in non-human primates have been consistent with the findings in rodents [4-6]. Recent research has focused on identifying the mechanisms underlying the anti-aging effect of CR. The first report on reduced mortality in rhesus monkeys was presented at the EB meeting last April [7]. A major goal of research into aging has been to discover ways of reducing morbidity and delaying mortality in the elderly [8,9]. The absence of adequate information on the effects of CR in humans reflects the difficulties involved in conducting long-term CR studies, including ethical and methodological considerations, amongst others.

Naturally occurring episodes of CR in human populations are not uncommon in some parts of the world. However, it is important to note that most of these populations are exposed to energy restricted diets of poor quality. They are often associated with short stature and late reproductive maturation [10], lower baseline gonadal steroid production in adults [11,12], suppressed ovarian function [13,14], impaired lactation performance [15,16], impaired fecundity [17] and impaired immune function [18,19]. The literature also indicates that low energy intake is often, but not always, associated with lower basal metabolic rate [17]. The pioneering studies by Keys and colleagues [20] found that severe CR induced changes in many variables including metabolic rate, pulse, body temperature and blood pressure. However, these diets were of poor quality as well and induced many adverse psychological effects.

A few studies have looked at the impact of CR on health and longevity with a high-quality diet. Kagawa [21] carefully analyzed Okinawan data documenting the incidence of centenarians on Okinawa Island is two to forty times greater than that of other Japanese communities. In these studies, the total energy consumed by school children was only 62% of the "recommended intake" for Japan. For adults, total protein and lipid intake was about the same, but energy intake was 20% less than the national average. Death rates from cerebral vascular disease, malignancy and heart disease on Okinawa were only 59, 69 and 59% respectively of those for the rest of Japan. In the 1970s mortality for people 60-64 yrs. of age was only half that of elsewhere in Japan. While these data are consistent with the hypothesis that CR increases life span in humans, there are possible other unmeasured differences, for instance genetic, between Okinawa and mainland Japan. To our knowledge, there is only one study [22] directly related to

the question of long-term CR in humans. Over a 3-yr. period, 60 experimental subjects received 2,300 kcal/day every other day, and on the other one liter of milk and 500g of fruit, giving a total average intake of 1,500 kcal/d vs. 2,300 kcal/d in 60 control subjects. Stunkard [23] reanalyzed this data and reported lower rates of admission to the infirmary (123 vs. 219 days) and a non-significant lowering of death rate (6 vs. 13) in those restricted vs. controls. Studies of dietary restraint in healthy normal-weight postmenopausal women did not show an association with a wide range of physiological, metabolic and health characteristics [24].

CR and Energy Metabolism

The first experiments of the effect of energy restriction in humans were in lean men by Keys et al in the 1950s[20]. In these classic experiments lean volunteers received 50% of their habitual intake. There was a decreased BMR when adjusted for body surface area (-31%), body weight (-20%) and for cell mass (-16%). However, there were indications of malnutrition with deficiencies in many micronutrients. Most of the other studies of the effect of energy restriction on energy metabolism have been performed in obese people. In several studies, a very low calorie diet resulted in a decrease in BMR, which was still significant when expressed per kg of body weight or per kg of fat-free mass [25-27]. A recent meta-analysis of studies in post-obese patients found a lower resting metabolic rate, even after adjustment for body size and body composition [28]. Careful studies of individuals who were formally obese have shown that energy turnover was approximately 15% lower than that of individuals of the same body composition who were never obese [29-32]. In one of these studies by Leibel et al, lean subjects were studied and clamped at a weight 10% below baseline. Energy requirement for weight maintenance was decreased by 10-15% even after adjustment for fat-free mass [29]. Part of this adaptation may be related to the cost of physical activity as elegantly shown by Weigle and Brunzell [32]. Therefore, there is evidence that a metabolic adaptation develops in response to CR and loss of weight in humans, in both obese and lean subjects.

Of relevance, we clearly identified a metabolic adaptation in five of the eight Biosphereians subjected to CR for two years during a stay in Biosphere 2 [33]. The subjects, measured within a week after the exit from Biosphere 2, had a decrease in adjusted 24-h energy expenditure and spontaneous physical activity in a respiratory chamber when compared to 152 control subjects. However, within the confinement of Biosphere 2, total daily energy expenditure measured by doubly labeled water was not characteristically low [33]. This was probably due to the relatively high PA (PAL=TEE/RMR=1.70 ± 0.06) required to harvest the food inside.

Energy metabolism in aerobic organisms is coupled to the generation of deleterious byproducts called reactive oxygen species (ROS). In fact, 2-5% of oxygen consumption is not used in oxidative metabolism of fuels but is associated with the production of highly reactive oxygen molecules such as the superoxide radical ($O_2^{\bullet -}$), hydrogen peroxide (H_2O_2), and the hydroxyl radical ($OH^{\bullet}$) [34]. Any increase in oxygen consumption increases electron (e^-) leaks, which represent the most important source of oxygen-containing radicals. $O_2^{\bullet -}$ is formed when an electron is added to an O_2.

In summary, there is evidence, particularly in the non-obese, for a metabolic adaptation in response to CR, and that the efficiency of weight maintenance is increased on the order of 10-15% compared to baseline body weight. Since core temperature is known to vary concomitantly with metabolic rate [35], we expect to observe a decrease in core temperature in response to CR.

CR and Oxidative Stress

The “oxidative stress hypothesis” of aging is supported by numerous observations: 1) Life span inversely correlates with metabolic rate in a wide variety of animals and is directly related to the amount of ROS produced [36]; 2) Over-expression of anti-oxidative enzymes or activation of defensive mechanisms against oxidative stress retards aging and extends life span in some organisms [37,38]; and 3) CR reduces oxidative stress, retards age associated changes and extends the maximum life span in various species

including mammals [3,39]. Thus, the amount of oxidative damage increases as an organism ages and is postulated to be one of the major causal factors of aging and therefore, of life span. Even if the mechanisms by which oxidative stress accelerates aging remain unclear, it is postulated that damages caused by ROS to protein, lipid, and DNA causes the age-related changes [39,40].

Protein oxidation by ROS produces abnormal protein modifications such as the formation of the carbonyl groups and the cross-linking which accumulates during aging in various tissues including eye lens, brain and hepatocyte [41]. One important end product of lipid peroxidation by ROS is lipofuscin, a pigment that accumulates with age in non-dividing cells such as neurons and muscle [42]. Isoprostanes are prostaglandin-like products of arachidonic acid peroxidation that have been putatively shown to be associated with oxidative stress [43].

However, most attention has been paid to the effects of ROS on DNA damage. ROS can induce formation of several base adducts in DNA, which are implicated in mutagenesis, carcinogenesis and neurological disorders [44]. Of major interest is the fact that the amount of DNA damage correlates with metabolic rate in various animals suggesting that ROS generated by aerobic energy metabolism may be a major cause of spontaneous DNA damage [45]. This idea is supported by recent studies on genetic analysis of human premature aging, pathological changes such as atherosclerosis, osteoporosis, and malignant tumors among others [44,46-49]. Syndromes of premature aging are associated with genes of the helicase family, which participate in DNA replication, transcription and/or repair.

An abundant form of DNA damage by free radical attack is 8-oxyguanine [8-oxoG][50]. Its formation can lead to G→T transversion mutations [51] since Adenine is misincorporated opposite 8-oxoG during DNA replication [52]. Free radical attack on DNA can also give rise to baseless, or apurinic/apyrimidinic (AP), sites. The most likely mechanism for this observation is that the DNA damage produced by oxidative stress results in destabilization of the N-glycosylic bond and the formation of an AP site. Moreover, the repair of 8-oxoG can give rise to AP sites as an intermediate in their repair. The presence of AP sites in DNA can be lethal to the cell [53], or cause mutations [53,54]. Notably, the formation of both 8-oxoG and AP sites increase with age [55,56]. Therefore, DNA damage from oxidative stress produced by energy metabolism is a potential cause of natural aging.

In the present study, we propose to measure protein carbonylation, isoprostanes as an index of lipid peroxidation, and DNA damage by the comet assay and the urinary excretion of 8-oxoguanine. We will test the hypothesis that CR will decrease damages to protein, lipid and DNA.

CR effects on CVD risk factors

Atherosclerosis is now recognized as an inflammatory disease [57]. The initiating event in the progression of atherosclerosis is believed to be the development of endothelial dysfunction. Potential causes of endothelial dysfunction include elevated levels of oxidatively-modified LDL; generation of free radicals (i.e. from smoking), hypertension, diabetes, and elevated levels of homocysteine. The injured endothelium responds to these various insults by developing procoagulant instead of anticoagulant properties, and by secreting a number of cytokines and growth factors. The release of these factors leads to the sequestration and accumulation of lymphocytes and macrophages from the blood and the migration and proliferation of underlying smooth muscle cells. Consistent with this paradigm is the recognition that in addition to the well-recognized CVD risk factors including lipids, lipoproteins [LDL cholesterol, HDL cholesterol, triglycerides], and blood pressure, other factors are of importance. Among those, hemostasis factors [factor VII, fibrinogen, plasminogen activator inhibitor-1], elevated levels of C-reactive protein (an acute phase protein and sensitive marker of inflammation), and elevated homocysteine are predictive of CVD events [58-60]. The increased hepatic synthesis of C-reactive protein is likely mediated by IL-6, which is in turn regulated by the pro-inflammatory cytokines TNF-α and IL-1 and is additionally synthesized by adipose tissue [61]. As evidenced below, CR has the potential to affect both the traditional CVD risk factors as well as the markers of inflammation.

Blood pressure is decreased by CR in the obese [62,63] and in chronically undernourished laborers [64]. Landsberg and Young have documented that CR is associated with a decrease in plasma norepinephrine concentration, decreased excretion of catecholamines, evidence of diminished sympathetic activity [65,66]. It is likely therefore, that the decrease in blood pressure during CR is mediated by a decrease in insulin concentration and sympathetic nervous activity [67]. Usually, CR does not affect total cholesterol or LDL cholesterol levels, as shown in studies by Wood et al [68] and Velthuis-te Wierik et al [67]. On the other hand, HDL cholesterol is significantly increased in proportion to the decrease in body weight [67,69].

CR may also influence the endothelial function of the vasculature and, therefore, protect against atherosclerosis. Recently, Perticone et al [70] reported in elegant studies that endothelial dysfunction often seen in obese or overweight subjects is due to oxidative stress [71-73] and can be reversed by acute administration of the potent antioxidant, vitamin C. It is therefore logical to hypothesize that CR will improve endothelial function in CR overweight volunteers, probably via a decrease production of ROS. We will measure endothelial function by measuring changes in the diameter of the brachial artery in response to reactive hyperemia following ischemia.

CR effects on insulin sensitivity and secretion

There is compelling evidence that CR and consequent weight loss in obese (diabetic and non-diabetic alike) greatly improves glucose metabolism by improving insulin action. In a comprehensive review, Kelley [74] concluded that weight loss in obese patients with type 2 diabetes mellitus not only reduces fasting hyperglycemia (reduction of post-absorptive hepatic a glucose production), but also increases insulin sensitivity (glucose uptake) in peripheral tissues (mostly non-oxidative glucose metabolism, i.e. storage). In a recent review, Ryan proposed that lifestyle modifications including body weight loss and increased PA provide health benefits and functional gains and should be promoted to increase insulin sensitivity [75]. Ross et al investigated the independent effect of equivalent diet-or exercise-induced weight loss and exercise without weight loss on insulin sensitivity in obese men [76]. The authors concluded that weight loss induced by daily PA without CR substantially reduced obesity and insulin resistance to level similar to that observed with diet induced weight loss. Whether a synergism between weight loss and PA exists is still debated. Most studies indicate that even in lean people, CR seems to increase insulin sensitivity. However, the most convincing data that long-term CR is an effective means of avoiding the development of insulin resistance occurring with aging are from monkey studies [5,6,77,78]. In lean humans, the most convincing data comes from results in the eight Biospherians who were exposed to a severe CR during most of the 2-year period inside Biosphere 2. In these 8 subjects, there was a clear decrease in fasting blood glucose and fasting insulin [79,80].

In summary, there is evidence that CR in obese and lean subjects alike improves insulin sensitivity. Whether the addition of exercise has a synergetic effect remains to be proven. Recently, Shulman has summarized the potential molecular mechanisms involved in the relationship between fat and insulin sensitivity [81].

CR effects on the activity of neuroendocrine axes and the autonomic nervous system

It is likely that the neuroendocrine system and the autonomic nervous system mediate many of the metabolic consequences of CR. The endocrine changes associated with caloric deprivation (CR or starvations) are well described in rodent models as recently reviewed Shimokawa [82]. Many of these alterations have been described in humans and include a fall in T_3 [83], an increase in cortisol secretion [84] and a decrease in gonadal function. It has long been hypothesized that the neuroendocrine system coordinates and integrates some of the anti-aging actions of CR [85-88], but little is known about the neuroendocrine pathways that are altered by chronic CR [89-93]. One of the major reasons for the paucity of data pertaining to CR and neuroendocrine axes is that neuroendocrine functions are difficult to study in

rodents. The present study in humans provides a unique opportunity to assess the neuroendocine axes in response to CR because collecting adequate blood samples is not a problem.

In a recent 48-h prolonged starvation study in mice, Ahima et al provided evidence that the induced reduction in leptin concentration caused a decrease in the activity of the gonadal and thyroid axes, and an increase in the activity of the adrenal axis [94]. The changes in activity of these axes during fasting could be prevented by leptin administration, suggesting a role for leptin as a master regulator of neuroendocrine status. These results support the “disposable soma theory” on the evolution of aging stating that longevity requires investment in somatic maintenance by reducing the resources available for reproduction [95]. Downregulation of neuroendocrine activity has been interpreted as a marker of somatic preservation [82] and leptin as the candidate endocrine mediator for this effect .

The role of leptin in neuroendocrine responses to fasting in humans has not been clearly proven; i.e. by administration of leptin during fasting. However, the available data is consistent with the somatic preservation hypothesis. In contrast to prolonged fasting, CR results in a decrease in leptin levels, but not its complete absence. For example, many highly trained endurance athletes have low leptin levels, but normal menstrual cycles. However, at equivalent fasting levels of leptin the loss of leptin diurnal variation is associated with menstrual irregularities, amenorrhea and increased cortisol levels [96,97]. Therefore, the diurnal rhythm of leptin may be more important in determining neuroendocrine function than the fasting concentrations. In this study, we will carefully characterize the plasma profile of leptin in response to CR and its relationship to the neuroendocrine and autonomic nervous systems. This will be achieved by measuring the diurnal changes in plasma concentrations of leptin, cortisol, growth hormone (GH) and thyroid hormones in conjunction with 24-h urine excretion of cortisol and norepinephrine. We do not anticipate dramatic changes in these parameters, which may remain within the physiologic ranges. For instance, the expected drop in T_3 and GH/IGF-1 may be within the lower part of the normal range, and the increase in cortisol within the higher normal range. However, these changes could play an important role in orchestrating the anti-aging effects of CR at the cellular level.

One area of discord between the human and rodent models of CR is GH. GH secretion is decreased in rodent models of CR, whereas humans that lose weight have an increase in GH secretion [98]. Obesity per se is associated with increases in free levels of IGF-1 [99,100] and FFA. FFA and IGF-1 both are known to decrease GH secretion through a negative feedback mechanism [101]. Weight loss decreases the levels of both FFA and free IGF-1 that should increase GH secretion. In contrast, leptin levels fall during CR, which should decrease GH.

CR effects on gene expression

Several groups have described changes in gene transcription related to senescence and reversal/prevention of these changes by CR in rodent skeletal muscle [102] and brain [103]. Several of these genes are dysregulated during aging in man [104]. Most of these genes function in energy-protein metabolism, as protein chaperones, or in oxidative stress pathways. We have selected candidate genes for study based on two criteria. First, genes were selected if they are dysregulated during aging and have an identifiable human homologue. Preference was given to genes that are normalized during CR in rodents. Second, genes were selected if they had been demonstrated to alter longevity in "lower" organisms and have an identifiable human homologue. We have generated a preliminary list of 49 genes which will be updated/modified as the field advances.

CR and Food and Macronutrients Preference

Studies in normal-weight subjects suggest that individuals with the greater preference for high-fat foods may be at high risk for significant weight gain [105,106]. This is likely to be the case for the overweight population we will recruit for this study.

Most individuals who lose weight are unable to sustain the weight loss, and data from our laboratory suggest that this is at least partially due to an unyielding preference for high fat/high sugar foods. Recently, we used the

Macronutrient Self-Selection Paradigm (MSSP©) and the Food Preference Questionnaire (FPQ©) developed in our laboratory [107] to compare fat preference and macronutrient self-selection in post-obese subjects with lean and obese subjects. We found that post-obese individuals were able to restrict total caloric intake to that observed in lean subjects despite no decreased preference for high fat/high sugar foods [108], a condition predisposing to weight relapse.

We will use the MSSP© and the FPQ© to assess overall fat/sugar intake as well as fat preference.

Physical Activity, CR and quality of life

Research unanimously supports the health benefits of PA in humans of all ages [109]. It is proposed that increasing PA alone, even in the absence of modifying other health behaviors, will improve quality of life and prevent the onset of chronic disease [110,111]. However, to date the only evidence that PA extends the life span is observational in nature [112]. It is extremely difficult to determine if increased PA alone decreases mortality because changes in PA are typically associated with changes in body composition and other health-promoting behaviors such as nutrition and smoking cessation, although most studies have adjusted for these potential confounders. Other studies of PA programs without CR have found that increased exercise is generally associated with improved mood, energy, and quality of life. Based upon these findings, we hypothesized that the combination of 12.5% calorie restriction and 12.5% exercise would yield the greatest increases in mood and quality of life and would be associated with the lowest rate of attrition.

HYPOTHESES

Caloric restriction (CR) from birth or during adult life in rodents and lower species prolongs life. Measurement of surrogate markers of longevity suggests that this may be the case in primates as well. The mechanism(s) responsible, and whether this is the case for humans is unknown. CR is associated with several well-known changes in metabolism, including lowering of the metabolic rate. It is unknown which, if any, of these adaptations might be responsible for extending maximum life span. One intriguing hypothesis is that CR lessens the oxidative damage and repair of vital tissues by reducing energy flux and metabolism. CR results in loss of weight and tissues, and lowers the rate of metabolism. A portion of this is the result of the reduced energy intake itself, and another portion is due to the decline in size of the metabolizing mass. Whether there is also a “metabolic adaptation,” defined here as a reduction of metabolic rate that is out of proportion to the decreased size of the respiring mass is a subject of continued debate.

We will test for this, and in addition, if the expected decline in metabolic rate (whether or not proportionate to the respiring mass) that follows CR is associated with reduced oxidative stress in tissues, and risk factors for age-related metabolic diseases, including cardiovascular disease and type 2 diabetes. In addition, we will test if combining physical activity (PA) and CR to produce the same caloric deficit alters the adaptations caused by CR alone. As part of these investigations, we will assess the expression of genes involved in energy metabolism and oxidative stress that are known to be associated with longevity in “lower” organisms.

This study is to test if chronic CR, as it does in rodents and “lower” species and possibly in non-human primates, improves surrogate markers of longevity in humans, and therefore might extend the maximum life span. Thus, this is mainly a descriptive undertaking. However, we also propose to test several interesting hypotheses.

- **Hypothesis A**. Chronic CR (resulting in loss of weight and maintenance of energy balance at a new lower body mass) is associated with several metabolic adaptations, including lower absolute and

relative rates of energy expenditure, lower body temperature, and evidence of lower tissue oxidative stress.

Aims: Measure and compare for difference:

A1) Energy expenditure (free living, sedentary 24-h, resting, exercise efficiency), and body temperature.

A2) DNA, protein and lipid oxidative damage.

- **Hypothesis B**. Chronic CR improves surrogate markers (risk factors) for chronic diseases, including cardiovascular disease and type-2 diabetes. These adaptations are the same whether the energy deficit is produced by combining PA and CR or by CR alone.

  Aims: Measure and compare for difference:

  B1) CVD risk factors (BP, lipid profile, hemostasis factors, homocysteine, endothelial function, and markers of inflammation).

  B2) Type 2 diabetes risk factors. (Insulin action and secretion)
- **Hypothesis C**. Chronic CR dampens the activity of the neuroendocrine axes, and lowers SNS activity, potentially by decreased leptin signaling. These adaptations are less pronounced when the same energy deficit is achieved by combining PA and CR.

  Aims: Measure and compare for difference:

  C1) Hypothalamic neuroendocrine function (thyroid, adrenal, and somatotrophic axes), and diurnal rhythm of leptin.

  C2) SNS activity
- **Hypothesis D**. Chronic CR is associated with adaptations in the expression of genes involved in aging, including those related to oxidative stress, energy metabolism (carbohydrate, lipid and protein), and longevity.

  Aims: Measure for differences in the expression of candidate genes in skeletal muscle and adipose tissue.
- **Hypothesis E**. Psychophysiologic outcomes are improved when the energy deficit is produced by combining PA and CR compared to CR alone.

  Aims : Measure for differences in weight loss, compliance, rate of drop out, quality of life (QOL), mood, cognitive function, fitness & strength, reaction times, physical activity & food records, and risk of developing an eating disorder.

CHARACTERISTICS OF THE SUBJECT POPULATION

Sixty subjects will be recruited for this study. Only subjects with a $25 \leq$ BMI < 30 and $25 \leq$ age < 50y for males and 25-45 for females will be included. The lower age limit for females is to avoid the confounding effect of menopause. We will make a conscious effort to enroll 50% males and 50% females. The ethnic/racial composition of the subjects will be proportional to that of Baton Rouge. We expect, therefore, to enroll approximately 20-25% African Americans in the presented study. Female subjects will be checked for pregnancy on a regular basis (each block of testing) and will be discharged from the study if pregnant. The following inclusion/exclusion criteria will also be followed:

<u>Inclusion</u>

- Healthy men and women
- $25 \leq$ BMI < 30
- $25 \leq$ age < 50 for males
- $25 \leq$ age < 45 for females (women age 40-45 will be screened for menopause)

<u>Exclusion</u>

- Personal history of CVD or elevated blood pressure (>160/90 mmHg)
- Personal history of diabetes
- Personal history of major psychiatric disorders
- Personal history of eating disorder
- Post obese (never have had a BMI > 32)
- Smoking
- Subjects exercising regularly (> twice per week)
- Regular use of medications except oral contraceptives
- Individuals with alcoholism or other substance abuse
- Pregnancy or lactation
- Abnormal EKG
- Individuals who have been resident in the greater BTR for < 1y
- Individuals in occupations requiring full alertness and motor skills (airline pilots, etc.) where even a small chance of postural dizziness would be unacceptable

<u>Psychiatric Disorders</u>: Participants will be screened using the Structured Clinical Interview for Diagnosis for the DSM-IV Axis I Disorders (SCID)[113]. Persons diagnosed with a lifetime history of psychotic disorders or Major Depression will be excluded. Subjects diagnosed with other psychiatric disorders will be excluded if it poses a threat to adherence to the protocol, as judged by the investigative team.

<u>Eating Disorders</u>: Participants will be screened for a history of anorexia nervosa, bulimia nervosa, and binge eating disorder [114] using the Interview for Diagnosis of Eating Disorders-Fourth Version (IDED-IV)[115]. Persons who are diagnosed with eating disorders (present or history) will be excluded from the study.

EXPERIMENTAL DESIGN

Our primary hypothesis is that long-term CR that is associated with weight loss and maintenance at a new and lower weight decreases absolute and relative (adjusted for body weight and composition) energy expenditure, and decreases tissue oxidative stress. We also hypothesize that creating the same energy deficit by combining CR and PA will blunt these metabolic effects, but provide similar results in the surrogate markers used to assess cardiovascular disease and type 2 diabetes mellitus.

Overall Description of the Experiment

The proposed study is a 6 mo. study to be completed over a period of 2 yrs. The study will involve 60 overweight healthy male and female volunteers divided into four equal groups matched for age and gender. As shown in figure 1, the four groups include:

<u>Group 1</u>: Controls (No CR but receiving a healthy diet as per the American Heart Association recommendations)

<u>Group 2</u>: CR (energy deficit 25%)

<u>Group 3</u>: CR 12.5% + PA 12.5% (energy deficit 25%)

<u>Group 4</u>: CR (LCD 800 kcal balanced liquid diet) followed after a 15% wt. reduction by wt. maintenance (“wt. clamping”).

Figure 1 ; 6- mo Experimental Design

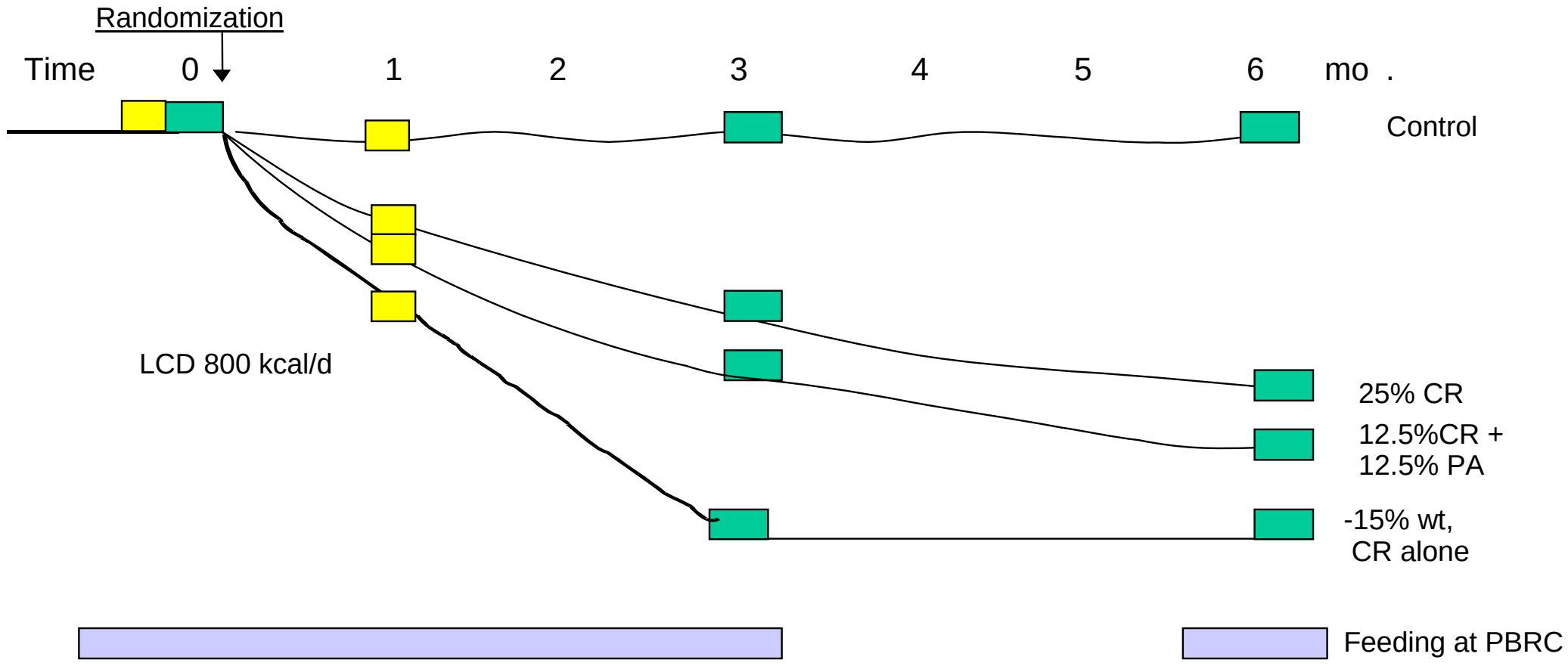

Green blocks denote metabolic test periods at baseline, 3 and 6 month periods (doubly labeled water plus 5-day inpatient stay (3 weeks total). Yellow blocks denote doubly labeled water measurements only (2 weeks).

This study is to test the feasibility and compliance to a 25% CR diet using several creative strategies, and to pilot and test several of the methods for a subsequent longer calorie restriction study. These include methods for assessing PA under free-living conditions, refinement of the assessments of the neuroendocrine axes, validation of the measure used to assess DNA oxidative stress, and final selection of the genes for tissue mRNA measurements. Because of the tight control of diet and exercise program, it will be impossible to blind all study personnel to the intervention assignment. However, most of the personnel performing the assessment measures (for ex. lab work, DEXA, energy expenditure etc.) will be blinded.

It is anticipated that compliance will be difficult for the volunteers and difficult to assess. Because of this, and in an attempt to improve the ease of compliance and to encourage it, all the volunteers will receive their meals at the Center for the initial 3.5 mos., and during the metabolic testing days. Compliance, however, will still be difficult to assess, since the measured parameters, including body weight, body composition, energy expenditure, dietary records, etc. are insufficiently precise or accurate to provide real-time objective information about it. Therefore, we have included a "weight clamped" group of subjects who have lost 15% of body weight by medically supervised LCD. This will allow the outcomes of interest to be assessed in a group of volunteers who will precisely maintain a new energy balance at an intake predicted to be 20-25% below their baseline energy requirements after rapid wt. loss (15%).

SCREENING OF SUBJECTS, DIET, EXERCISE REGIMEN AND BEHAVIORAL INTERVENTION

Screening paradigm

Following a brief telephone interview, prospective volunteers will be invited for a series of 3 screening visits to the Center**.**

Screening visit one includes explanation and signing of the informed consent. At this visit volunteers will have height, weight, and blood pressure recorded, and complete a brief personal and family medical history and eligibility questionnaire. Potential participants will be interviewed by the psychology staff using the SCID and IDED-IV to identify people who have a history of psychosis, major depression, eating disorders, or another psychiatric problem that might adversely impact participation in the study. Also, the psychology staff will assess motivation to change dietary and exercise habits and to attend feeding and counseling sessions. Potential volunteers will be administered an adaptation of the Motivation and Readiness Scale to measure intention to adhere to various aspects of the intervention. Furthermore, they will be interviewed regarding potential obstacles to participation in the study, e.g., work schedule, family responsibilities, travel schedule, and driving distance to PBRC**.** A demographic questionnaire will also be administered.

Screening visit two includes a physical exam, ECG, and distribution of dietary/PA questionnaires**.**

Screening visit three includes urine and blood samples for CBC, chemistry panel with electrolytes, urine analysis, and careful review of the diet and PA diaries.

Between screening sessions, participants will be instructed to self-monitor all foods they consume and all exercise sessions for two weeks. Structured self-monitoring booklets will be provided to facilitate the completion of this task. To be eligible for the study, participants must complete and bring (to session 3) the self-monitoring records for at least 12 of 14 days (85%). Participants failing to meet this criterion will be provided an additional two weeks to complete this task. If they fail to meet the requirement of this "behavioral run-in", the participants will be excluded from the study. Using these rigorous screening criteria, only highly motivated persons, with few obstacles to participation will be enrolled in the study. If all of the above visits are completed successfully, the potential volunteer will be invited to participate in this study and will have a final session to discuss all the study requirements. Subjects will then enroll in the dietary portion of the baseline study and undergo 19 days of testing. At the end of the baseline-testing period, volunteers will be randomized to one of the four groups (see Figure 1).

Diets and diet delivery

Volunteers will receive a diet based on the American Heart Association guidelines, providing 30% of the calories from fat, 55% from carbohydrate and 15% from protein [116]. The diets will consist of three meals and an evening snack arranged in a 5-day rotating menu that will vary as the study proceeds. All foods will be prepared and measured by the Pennington metabolic kitchen staff. The volunteers will be provided with all of their meals by the Metabolic Kitchen, and will be encouraged to consume all food provided. The volunteers will be required to consume breakfast and dinner at the PBRC dining facility on weekdays. Weekday lunches and snacks will be packaged for take-out and will be distributed at breakfast and dinner. Weekend meals will be packaged and distributed at the Friday dinner. In our experience, most volunteers find a 30% fat diet quite filling, since they usually eat a high fat diet with a much lower food volume. We will include bulky foods such as salads and whole grains. Since the volume of food plays an important role in satiation [106], we anticipate that this diet will limit the feeling of hunger during the CR. On the other hand, it may prove difficult to maintain body weight in subjects enrolled in the control group. During the baseline period volunteers will be started on the energy level that most closely matches their estimated energy requirement according to the Schofield equation for RMR and an estimate of PA level as per WHO [117]. Each study diet will be prepared at five energy levels (1800, 2200, 2600, 3000, and 3400 kcal/day). For energy levels between these values, subjects will receive 100 kcal "unit" foods to include cookies, breads, and muffins, meeting the nutrient specification of the diet. Subjects will be allowed limited choices of seasonings and beverages within their assigned dietary treatment.

Beverages containing caffeine will be limited to less than 5 cups per day. Alcohol will not be served at our facility, but will be allowed in moderation. Subjects will be encouraged to record any deviations from the diet and the consumption of self-selected food and beverage items in a food diary.

After baseline testing and randomization, the target calorie level for each participant is determined. This is achieved by measuring energy expenditure by doubly labeled water (twice at baseline, once whilst the participant is consuming usual diet at home, and once whilst consuming meals provided by PBRC metabolic kitchens) and by the metabolic chamber, baseline feeding requirements and the assigned study intervention. The LCD participants will be fed 800kcal/day regardless of energy requirements until 15% weight loss is achieved. Following this the "weight clamped" subjects will be fed or prescribed a similar diet providing the necessary energy to maintain the 15% wt. loss. Subjects receiving less than 1800 kcals/day will be provided with a multi-vitamin supplement to ensure adequate nutrition is maintained during energy restriction. This will be monitored by pill counts each month.

Menu Development. Menus are developed by the metabolic kitchen research dietitians, and are analyzed using the MENu (Moore's Extended Nutrient) database. Recipes are selected to include regional food preferences to increase dietary adherence. The research dietitian reviews the finalized menu with each potential participant before the research project begins. The participant's food likes, dislikes and intolerances, including food allergies, are discussed. If necessary, foods that a participant refuses to eat are substituted with an equivalent food item. Individual choices, within study limitations, are given for beverages throughout the study.

Diet Distribution. Daily food production sheets for each participant are used, listing day, menu cycle, meal, food items required with portion weights, and special dietary requirements. When preparing the meal trays, the food production sheets are followed. Additionally, foods are labeled for participant identification. Foods are placed on individual meal trays until served, or are individually packaged for take-out, following tray assembly forms. At the time of meal pick-up, the hostess reviews the menu with the participant, checking off the foods to confirm all are provided. Meals are served to the participant on test days only after all study procedures have been completed. A meal ticket is given to the participant by an authorized nurse or scientist, which is presented to the hostess.

Each participant completes a Daily Food Diary to assist with compliance assessment. The participant is asked to record study foods not eaten, non-study foods eaten, beverages consumed, number of unit foods eaten, and other study-specific food consumption information. The dietetic coordinator reviews the Daily Food Diaries and compiles a compliance score for each participant. Additionally, the dietetic research associates obtain daily comments from each participant, and record dietary progress notes. Potential problems with meal acceptance are identified and resolved. All staff members throughout the study provide personal attention and encouragement to continue on the diets. The first 3.5 mos. of complete feeding at the Center will be used to teach volunteers to comply with the diet while in free-living feeding.

Low-Calorie Diet. Creating a 15% wt. loss in overweight individuals is relatively unexplored. Drawing from the obesity literature, an 800-kcal/d diet with 70g of high quality protein delivered in calorie controlled liquid portions is likely to give optimal weight loss with minimal hunger. Since subjects with a BMI between 25 and 30kg/m^2 will have a smaller caloric deficit than obese, it may require up to 10 weeks to reach a 15% wt. loss.

The main safety concern with very low calorie diets is a life threatening cardiac arrhythmia. This complication was first reported in young healthy women following 300-400 kcal/d liquid diets for an average of 5 mos. [118]. At autopsy the hearts of these women resembled the protein-depleted hearts of

victims who did not survive concentration camps. Since lean subjects conserve protein less efficiently than the obese, one must take precautions to avoid this complication [119,120]. Further studies have shown that life-threatening arrhythmias are preceded by a drop in QRS voltage followed by a prolongation of the QT interval [121]. A drop in QRS voltage has been seen only in diets of less than 600-kcal/d [122]. Since one would expect an increase in QRS voltage with weight loss, a drop in voltage should be a sensitive test to screen for nascent cardiac toxicity of the diet [123]. Gallstones are also a potential risk with rapid wt. loss [124]. This risk, however, appears to be related to gallbladder stasis that can be avoided by including 10g of fat with one meal each day [125].

Subjects will be placed on an 800-kcal/d diet divided into 5 liquid portions (HEALTHONE). The diet will contain 70g of high quality protein, RDA of vitamins and minerals, 10g of fat with one meal and the remaining macronutrients as carbohydrate. Like the other 3 groups, subjects in this group will come to the metabolic kitchen every weekday to consume breakfast and dinner. The other liquid portions will be distributed daily to be consumed at home. Weekend meal replacements will be distributed Friday evening. Subjects will be seen in the clinic for measures of diet adherence, postural vital signs and metabolic weight once per week. Blood will be drawn and an electrocardiogram performed every 2 wks. If the chemistry panel shows potassium level below normal range, supplementation will be given. Upon achieving a 15% wt. loss, subjects will be progressively put back on solid food (same diet as other groups) in order to maintain the wt. loss. Subjects not attaining a 15% wt. loss by 14 wks. will be judged non-compliant and discharged.

Exercise

Subjects assigned to this arm will perform regular exercise at an energy cost of 12.5% of total daily energy needs measured at baseline by doubly labeled water, 24h-chamber and intake/balance requirements. The exercise regimen will consist of 5 days/wk. sessions of either walking/running or bicycling, or a combination (2 days). The energy cost of the exercise sessions will be calculated from the weekly desired energy cost divided by five. We have selected 5 rather than 7 days per wk. of exercise to comply with the American College of Sports Medicine (ACSM) recommendations of 3-5 days per wk. of aerobic exercise [126]. Five days per wk. is thought to be the upper limit to minimize the risk of injury [127]. Based on experience, we feel that the subjects' compliance and enjoyment of the PA will be greater if they are allowed to choose from several modes of exercise.

The exercise prescription for each subject will be individually developed and assigned. Since the goal of the exercise session is simply to "utilize energy", subjects will be allowed to choose a comfortable exercise intensity within the range recommended by the ACSM (i.e., 50-75% of maximum oxygen uptake or 60-85% of maximum heart rate)[126]. Briefly, the time subjects will need to walk, run or bicycle will be calculated from oxygen cost of activities measured in the exercise facility. Target heart rate will be the average heart rate measured during the steady-state measurement. Subjects will be instructed to exercise for the prescribed amount of time at an intensity that maintains a heart rate within 5 bpm of target heart rate measured via a portable heart rate monitor. Individual exercise prescriptions will be readjusted weekly for the 1st mo.; every 2 wks. for the next 2 mos.

Following the initial testing period, subjects randomized in the CR+PA arm will begin their exercise program (training phase) slowly (so as not to induce soreness or injury) and in coordination with the diet. The goal, however, is to have the subjects achieve their exercise prescription in its entirety by 6 wks. (Table 1). Subjects will exercise between 40 min. (jogging at 6.0mph) and 95 min. (walking at 3.5 mph) per session depending on the mode and intensity of the chosen exercise.

Table 1

Week	Prescription
1	2 d/week for ½ calculated time
2 and 3	4 d/week for ½ calculated time
4	4 d/week for ¾ calculated time
5	4 d/week for full time
6	5 d/week for full time

The PBRC Fitness staff will have frequent contact with the subjects. During the 6-wk. training phase, subjects will perform all exercise at the PBRC Fitness Center, closely monitored by the fitness staff. After this progressive training phase, subjects will exercise at the PBRC 3 days per wk. and have the option of exercising on their own for the remaining 2 days. Since the terrain around Baton Rouge is flat, subjects will be allowed to walk or run outside, but will be required to bicycle indoors on a cycle ergometer due to a greater difficulty of controlling environmental factors (i.e., wind, traffic, and road surfaces). Exercise compliance will be strictly monitored with exercise logs, heart rate data and body wt. Subjects will also be monitored using accelerometers and doubly labeled water at different times. Thus, subjects may be asked to exercise more frequently at the Center if compliance to the program is in question.

Timeline

<u>During feeding phase (-.5 to 3 mo.).</u> During the first 3.5 mos. of feeding at the Center, participants will meet as a group each week to discuss: a) basic concepts related to the participant's, b) problems related to compliance and attendance that participants are experiencing, c) preparation for the self-selected diet period and exercise regimen (in 1 arm), and d) basic principles of lifestyle behavior modification. These groups will attempt to boost morale and motivation to comply with the feeding protocol and will provide psychoeducational material that prepares the participant for the next phase. Participants will be asked to fill out a daily food record during this period.

<u>During Mo. 4-6.</u> During the next 3 mos., participants will learn to modify eating behavior (and PA in 1 arm) while eating a self-selected diet (CR or CR+PA). During the previous phase, the concepts of lifestyle behavior modification will have been presented. During this phase, these concepts will be translated into action. The primary aims of this phase will be to: a) follow the prescribed dietary plan, b) follow the prescribed PA plan, c) eat 3 meals per day on a regular schedule, d) solve problems that cause sub-optimal compliance with the program, and e) manage emotions or other states (e.g., hunger) that cause sub-optimal compliance. To accomplish these objectives, participants will attend individual or group therapy sessions every 2 weeks. Participants will be asked to fill out a daily food record during this period.

<u>Individual tailoring of treatment.</u> One aspect of lifestyle behavior modification, i.e. frequent and regular therapeutic contact, has been found to be very important for long-term behavior change. To accomplish frequent contact, treatment will be tailored to the needs of the individual participant using internet-based contact and/or telephone contact. Also, for persons who are having difficulty following the prescribed dietary or PA program, the intervention will be tailored to address the unique problems of the individual. The rate of predicted weight loss will be calculated using a 2-reservoir dynamic model of the energy balance equation as described by Alpert [128]. This data will be used to monitor compliance and will not be shared with the volunteer. Metabolic weight will be measured once per week. Participants who fail to meet 60% of the expected rate of wt. loss will be interviewed very carefully to identify reasons for potential sup-optimal compliance with the prescribed treatment. Compliance will also be assessed by measures of metabolic rate, heart rate monitors, accelerometers and doubly labeled water. If problems

related to compliance are identified, solutions to the problem will be established and individualized interventions will be initiated and tested for specified periods of time. If these interventions result in hypothesized changes in weight, they will be continued; if not, new strategies will be formulated and tested until success is achieved. Only after different tries, subjects will be asked to leave the study.

Outcome measures

Primary outcome. The primary endpoint will be the rate of energy expenditure under resting, sedentary and free living conditions. Each component will be assessed by state-of-the-art methods (ventilated hood, respiratory chamber and doubly labeled water). Sedentary energy expenditure in the respiratory chamber has been used for the power analysis. The reason is that this method is the most sensitive for assessing all of the components of daily energy expenditure, except for the cost of voluntary activity under free living conditions, and is therefore the most likely to uncover differences in energy expenditure for weight maintenance induced by chronic CR. As shown in Table 2, 5-11 subjects are sufficient to detect a 10% decrease in absolute or adjusted 24-h energy expenditure. Furthermore, assuming an intra-subject variability of 7.5%, a 15% decrease in total daily energy expenditure can be detected by doubly labeled water with only 11 subjects per group. With 15 subjects enrolled per arm in the study, we feel confident that we will have the necessary power of detecting significant decreases in both sedentary and total daily energy expenditure.

Secondary outcomes. While the proposed studies have sufficient power to detect differences for the primary endpoint, because of the novelty and opportunity offered by this study, we also plan to monitor several secondary outcomes of interest in a more descriptive manner. However, the methods and number of subjects may not always be up to the task of uncovering suspected differences.

1. *Changes in body composition*. We hypothesize that approximately 2/3 of wt. loss during CR will be fat mass, but more (75%) when a combination of CR and increased PA is the cause of similar negative energy balance. Body composition will be measured by DEXA, the new gold standard. Because of the precision and reliability of the method, small differences in changes in fat mass and fat-free mass can be detected.
2. *Changes in oxidative stress*. At baseline and at different phases of the study, oxidative stress will be measured indirectly by DNA damage, lipid peroxidation (isoprostanes), and protein carbonylation.
3. *Changes in neuroendocrine activities*. The thyroid, somatotropic, and adrenal axes will be measured over 24 hrs. by blood sampling and compared to changes in leptin profile. Activities of the thyroid and somatotropic axes are hypothesized to decrease in response to CR, whereas the adrenal axis will increase. Differences in areas under the curve will be detectable with our number of subjects.
4. *Sympathetic nervous activity* is predicted to decrease in response to CR (spectral analysis of heart rate and urinary excretion of catecholamines).
5. *Changes in cardiovascular disease risk factors and insulin sensitivity/secretion.* We hypothesize that both CVD factors and insulin sensitivity will improve in a similar manner in the groups subjected to CR alone or a combination of CR+PA. Insulin secretion is not expected to change.
6. *Changes in tissue gene expression*. We will measure these changes in muscle and adipose tissues by quantitative RT-PCR. The aim of this endpoint is to test the hypotheses that CR alters the pattern of gene expression in adipose tissue and skeletal muscle such that: a) oxidative stress is reduced; b) energy is conserved, and; c) "ancient" genes associated with longevity are regulated in a fashion consistent with involvement in longevity from animal data.

7. *Changes in quality of life*. We hypothesize that subjects enrolled in these studies of CR or CR+PA will improve their quality of life because of weight loss. However, subjects enrolled in the CR+PA group will have a better quality of life than those in the CR alone group.

METHODS AND PROCEDURES.

Psychological Assessment measures

Most of the questionnaires described below are attached in an Appendix.

Psychiatric Disorders at Screening

Structured Clinical Interview for Diagnosis of DSM-IV Axis I disorders (SCID)[113]. The SCID is an interview assessing all of the primary psychiatric disorders as defined by DSM-IV (Amer Psych Ass, 1994[114]). A trained fellow in psychology will conduct the interview, and Dr. Williamson will make diagnoses (Appendix V).

Assessment of Eating Disorder Symptoms

Safety of the CR procedures is one of the crucial questions to be addressed in this research project. Voluntary dietary restriction is considered to be a common precursor to the development of eating disorders [129], though there is considerable debate about the significance of this risk when advising overweight adults [130]. To objectively assess eating disorder symptoms throughout the study, 3 measures were selected, together providing a comprehensive approach for measuring the safety of CR, as it pertains to eating disorders.

Body Shape Questionnaire *(*BSQ)[131]. The BSQ will be used to measure concern with body size and shape, a common feature of sub-threshold eating disorders. The questions of the BSQ are not face valid as indices of eating disorders, which makes it a good screening measure. The BSQ has been found to be one measure that can predict the onset of eating disorder symptoms [132]. Participants who score greater than 100 will be excluded at baseline. Participants who score greater than 120 at any assessment point of the experiment will be considered for termination from the study (Appendix V).

Interview for Diagnosis of Eating Disorders, version 4 (IDED-IV)[115]. The IDED-IV will be used to screen for the presence of eating disorders at baseline, an exclusion criterion for the study. The IDED-IV is the only interview method that has been validated for the diagnosis of anorexia nervosa, bulimia nervosa, and binge eating disorder. The IDED-IV will also be administered at 6-mo intervals to assess for the development of eating disorders. Persons who report 4 of the 8 combined symptoms of anorexia or bulimia nervosa will be excluded.

Multiaxial Assessment of Eating Disorder Symptoms (MAEDS)[133]. The MAEDS is a self-report inventory that measures the six symptom domains related to eating disorders: binge eating, restrictive eating, purgative behavior, fear of fatness, avoidance of forbidden foods, and depression. Scores on all scales are standardized using t-scores. Participants who score greater than a t-score of 70 (two standard deviations above means for a normative sample) on 3 or more scales will be excluded at any phase of the experiment.

Three Factor Eating Questionnaire (TFEQ): TFEQ is a 56-item measure that assesses three aspects of eating and hunger. The Restraint scale measures current dietary restriction. The Disinhibition scale measures the extent to which the participant eats in response to external (e.g., emotional, social) triggers.

The Perceived Hunger scale provides a global level of hunger and is correlated with the Disinhibition scale.

Revised Restraint Scale (RS). The RS is a 10-item questionnaire designed to assess eating patterns of eaters who are currently attempting to restrict their dietary intake. The scale is reported to have adequate reliability and validity among normal weight subjects, accurately predicting different eating patterns. Scores less than 15 defined unrestrained eating; scores 15 and greater defined restrained eating.

Dieting Questions. Current dieting is defined as a current effort to restrict caloric intake to lose weight. Three questions have been validated as indicators of current dieting, with a high level of internal consistency (alpha = .85).

Dutch Eating Behavior Questionnaire (DEBQ). The DEBQ is a 33-item measure designed to assess current dietary restraint and the tendency to overeat in response to negative emotions or external cues. Normative data are available according to gender and weight status.

Assessment of Quality of Life, Mood, and Psychopathology
Measures of quality of life, mood, and psychiatric symptoms will be administered at baseline and at each assessment point.

General Quality of Life. The Satisfaction with Life Scale will be used to measure overall quality of life. This 5 item scale has been found to be a reliable and valid indicator of quality of life [134]. It will be administered at baseline and at 6-mo. intervals to assess changes in quality of life. This brief measure has been used in many studies with many different populations [135]. Also the RAND 36 item Health Survey (SF-36)[136] will be administered to assess eight health outcomes related to CR, e.g., energy/fatigue, emotional well being, and general health perceptions. General Health indicators, e.g., somatic symptoms and social dysfunction, will be measured using the 28 item General Health Questionnaire (GHQ)[137].

Depression. Symptoms of depression will be measured using the Beck Depression Inventory II (BDI-II)[138]. The BDI-II can be used to assess current levels of depression (past two wks.)(Appendix V).

Psychopathology. General psychopathology will be measured using the Symptom Checklist 90 (SCL-90)[139]. The SCL-90 measures 9 psychiatric symptom domains and 3 general indices of psychopathology. It can be used to measure current psychopathology (Appendix V).

Assessment of Cognitive Function
At each assessment period, cognitive functions (memory, attention, and concentration) will be objectively evaluated using 4 tests selected to measure relatively subtle changes in cognitive functions possibly associated with CR.

Auditory Consonant Trigrams (ACT)[140]. The ACT is a sensitive measure of attention and memory to auditory verbal stimuli. It has been validated as a measure of prefrontal lobe functioning [141].

Conners Computerized Continuous Performance Test (CCCPT)[142]. The CCCPT is a computerized reaction time test for visual stimuli. It is very sensitive to changes related to attention and concentration.

Rey Auditory Verbal Learning Test (RAVLT)[143]. This verbal learning test uses a fixed order word list format. Immediate and delayed recalls are measured. Also there is a recognition memory task in the RAVLT. There are alternate forms for the test, which allows for use in a longitudinal study.

Benton Visual Retention Test (BVRT)[144]. The BVRT is a memory test that has alternate forms and can be used in longitudinal studies such as the proposed study. The BVRT uses nonverbal visual stimuli to assess immediate and delayed recall.

At baseline and at different time periods (see Figure 1), tests will be conducted on our inpatient metabolic ward (4.5 day admission) using the scheme presented in Figure 2 and the following methods.

Figure 2: Testing block (4.5d admission at PBRC from days 14-18)

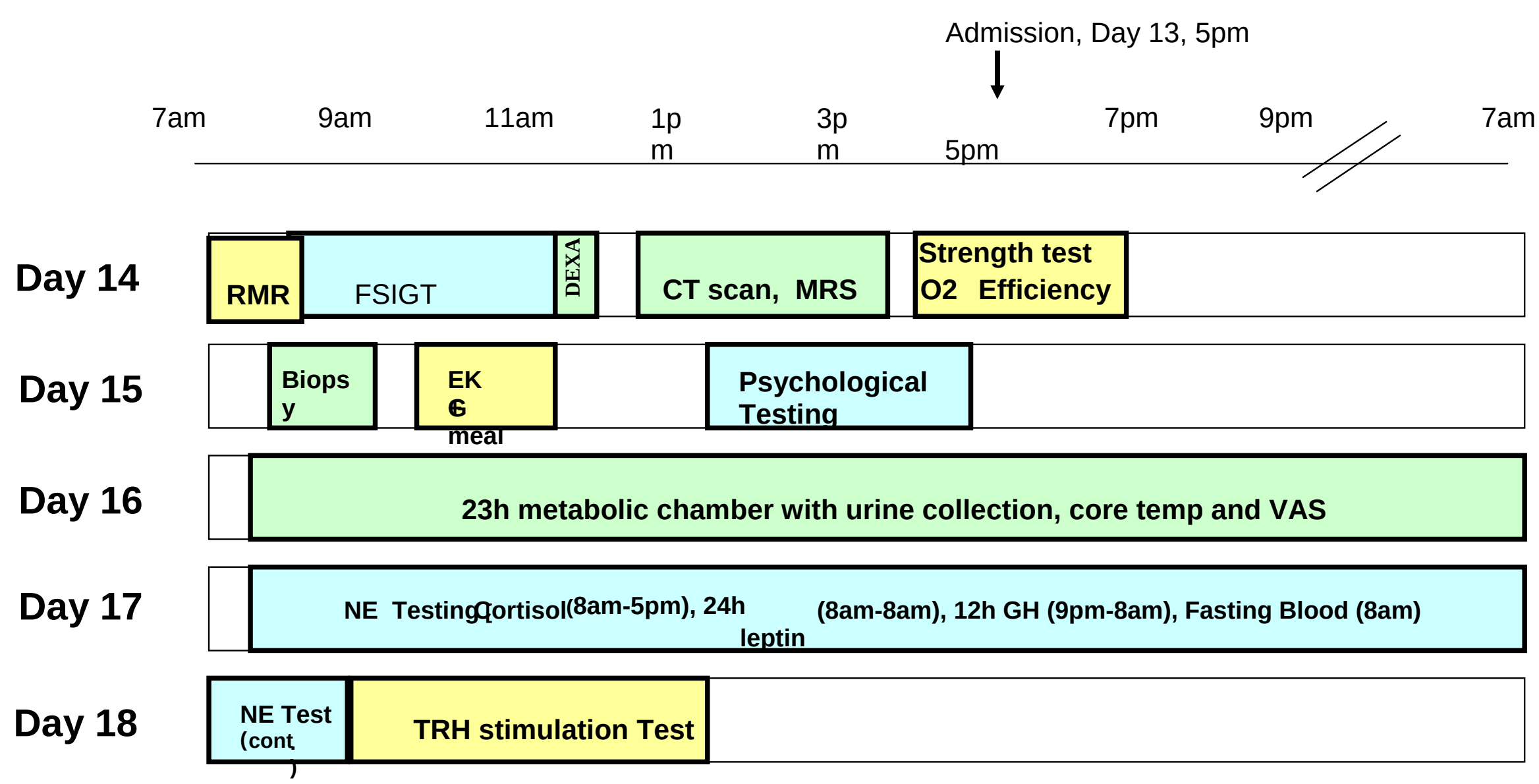

The measure of energy expenditure by doubly labeled water will be performed from days 1-14. The VO2 max, DEXA, fat selection (MSSP) and brachial artery tests will also be performed during that period as an outpatient. The order of tests in this figure is representative only. Test order may change if the availability of equipment changes.

Body composition and bone mineral density.

DEXA scans will be performed using a new Hologics QDR 4500A whole-body scanner. The protocol requires that subjects lie on a table wearing a hospital gown and no metal containing objects, while the scanner emitting low energy X-rays, and a detector passes along the body. The scan takes less than 4 min. and the radiation dose is less than 1 mrem, equal to about 12-h of background radiation. The scans are analyzed with the latest software QDR for Windows V11.1. Using our old instrument (QDR 2000), coefficients of variation (CV) for body composition measurements of lean mass, fat mass and percentage of body fat were 0.8%, 1.6%, and 1.7%, respectively. We will also use the DEXA data to estimate muscle mass using a method developed by Dr. Heymsfield's group [145].

Bone mineral density of the hip and proximal femur will be measured on the same scanner in a matter of mins. Subjects lie supine and motionless while the DEXA scanner moves along the length of the subject's left femur and lasts approximately 2 min. for a 6-inch length. The hip regions of interest are the femoral neck, ward's triangle, greater *trochanter,* and inter-trochanter region. Area, BMC, and BMD are all measured. For the most part, the analysis is automated, with Ward's triangle being automatically located by the system, because this is the area of initial bone loss in the femoral neck. There is a reference database for comparing BMD vs. age. Potential changes with intervention will be assessed.

Computed tomography
Abdomen for fat distribution. A single cross-sectional, 1cm width, scan is obtained centered at L2-L3, using 170mA, a scanning time of 1 s, and a 512 x 512 matrix (GE Light Speed, General Electric, Milwaukee, WI). Using commercially available software, areas of bone, adipose tissue and skeletal muscle are measured electronically by selecting regions of interest defined by attenuation values: ~200 HU (Hounsfield units) for bone, -30 to -190 HU for adipose tissue, and 0-100 HU for muscle. Mean muscle attenuation is determined from all pixels within this range [146-148].

Thigh and calf for muscle lipid infiltration. A single 1-cm thickness cross-sectional scan will be obtained of both the mid-thigh and mid-calf will be obtained by computed tomography (GE High Speed, General Electric, Milwaukee, WI). The 3-second scan will be performed at 120 kV (peak), 100 mA, to measure thigh lean tissue and adipose tissue cross-sectional area, composition (lean tissue) and distribution (adipose tissue). Subjects will be scanned while supine. The field of view is 32 cm and the matrix is 512 x 512 pixels. A range of attenuation values will define regions of interest for measurement of tissue areas. Thigh adipose tissue is electronically measured by using commercially available CT software (GE Medical Systems, Milwaukee, WI), with the region of interest window set to attenuation values of -200 to -1 Hounsfield units (HU).
To determine adipose tissue distribution, the fascia lata will be identified and outlined by manual cursor and regions of interest, set to the attenuation range for adipose tissue, will be used to measure the cross-sectional area of adipose tissue above and beneath the fascia lata.

Spectral Magnetic Resonance Procedure (MRS). A magnetic resonance scan will be performed at the Neuromedical Imaging Center on Hennessy Blvd in Baton Rouge. During the sMR experiments, subjects will be asked to lie supine on the patient table of the 1.5 magnet for approximately 45 min. Measurements will be obtained from the calf with the subject's leg positioned inside a radio frequent (RF) ^{1}H coil. Because the degree of knee extension and the angle of foot rotation can affect the quality of the spectra (i.e the amount of splitting between the IMCL and EMCL peaks), the actual positioning of the lower leg will be determined. Foam pads and pillows will be used to ensure the subject will rest comfortably during the testing. The subjects will be informed that the imaging scans will produce loud "knocking" noises and a headset with music will be provided to mask and minimize this noise.

Exercise monitoring and VO_{2max}
Exercise monitoring. Subjects will be instructed to maintain their PA and exercise habits during the 6 months. of intervention unless they are randomized to the CR+PA arm. In this arm, subjects will be instructed to comply with their almost daily exercise prescription. PA levels will be monitored for one week each month by providing each subject with their own portable heart rate monitor and accelerometer and regularly (twice weekly) downloading 24-h data from these free-living monitors. Uni-axial accelerometers will be used to monitor free-living PA [149,150]. The Stanford 7 day physical activity recall will also be administered once per month to all subjects to monitor any change in PA levels.

In addition, free-living 24-h energy expenditure (doubly labeled water) will be measured periodically throughout the study and will serve as an additional insurance. Subjects will be blinded as to when the doubly labeled water tests will be performed. This will be achieved by "dosing" the subjects with either tap water or doubly labeled water every 2 weeks. The participants will also provide urine samples each week. In Phase 1 of CALERIE actual dosing with labeled water will occur twice at baseline, at one month and at 3 and 6 months. Subjects who appear to have changed their exercise and/or PA patterns (compared to baseline) will be interviewed and the situation corrected. Subjects who fail to comply with these constraints will be further counseled and compliance strategies will be discussed.

Whole-body maximal aerobic power (VO_{2max}) and strength VO_{2max} will be measured by a progressive treadmill test to exhaustion using standardized procedures. Testing will be performed in the morning after an overnight fast, using a modified Bruce protocol, which has been used successfully in sedentary and endurance-trained subjects at a variety of fitness levels [151,152]. During the test, O_2 consumption and CO_2 production will be measured continuously by indirect calorimetry and heart rate will be recorded. A physician will be present for measures in subjects who are older than 45 or have at least one of the risk factors as described by the ACSM guidelines for exercise testing and prescription [153]. These subjects will be monitored using a 12-lead EKG.

Muscle strength and endurance will be measured via isokinetic testing on a Cybex machine during knee extension and flexion. Isokinetic testing allows for measurement of force velocity or power velocity curves as well as peak force and peak power. Unlike other methods of measuring strength, isokinetic testing is quick and less likely to cause injury in subjects not used to performing this type of testing. Before isokinetic testing, subjects will have an introductory trial to the procedure for familiarization with the task. The procedure involves an initial test of 3 repetitions at 60 degrees/sec. to measure peak force and power followed by a test to fatigue at 180 degrees/sec.

Energy expenditure

RMR. Indirect calorimetry will be performed before the assessment of insulin sensitivity/secretion using a Deltatrac II Metabolic cart (Datex-Ohmeda, Helsinki, Finland) over a 45-min. period. The analyzer will be calibrated before each study with standardized gases containing 5% CO_2 and 95% O_2. After quietly resting for 5-10 minutes, a transparent plastic hood connected to the device will be placed over the head of the subject. Calculations of O_2 consumption and CO_2 production will be made from continuous measurements of CO_2 and O_2 exchange over 45 minutes. Subjects will be asked to remain motionless and awake during the test and the last 30-min. of the measurement will be used to calculate RMR. The rate of protein oxidation will be determined from the rate of urinary urea production and the rates of carbohydrate and lipid oxidation according to Elia and Livesey [154,155].

Metabolic Chamber. 24-h energy expenditure and substrate oxidation will be measured in a metabolic chamber at baseline and at each block of testing. Volunteers will enter the chamber at 7:45 AM after an overnight fast, and leave the chamber at 7:00 AM the next morning. During their stay in the chamber, no exercise will be allowed. Meals will be served according to a fixed schedule. Microwave motion detectors provide continuous monitoring of the volunteers' spontaneous physical activity. The 2 metabolic chambers, each of which measure 10'x14'x8', corresponding to a volume of 27,000 L, were designed to provide a pleasant ambiance for study volunteers. The rooms have two windows, and are furnished with a bed, desk and chair, television, radio/tape player, telephone, microwave, sink and toilet with privacy curtain. Once a mo., the accuracy and precision of the calorimeters are assessed by 24-h propane combustion tests.

The Pennington Metabolic Kitchen staff will prepare all food consumed in the chamber. At baseline, the energy content will be estimated according to a previously developed equation and adjusted during the day on the basis of the energy expenditure of the first 7 hrs. of measurement [156]. After the baseline recording, energy content of the food for the subsequent chamber measures will be calculated as 100%, 87.5%, or 75% of baseline 24-h measured energy expenditure, according to the study arm. 24-h urine will be collected for norepinephrine and cortisol excretion rates determinations. During each stay in the chamber, subjects will be asked to fill visual analogue scale (VAS) to measure hunger, satiety, fullness and desire to eat during the course of the day. These VAS tools, first developed by Blundell have been fully validated [157] and used in association with plasma leptin changes in relation to daily changes in hunger and satiety [158]. Core temp. will be measured and recorded every 5-min. during the 24 hrs. of energy expenditure measurements in the chamber. At 7:00 AM the volunteers will swallow a 10 x 20-mm silicone coated radio-capsule (Human Technologies, Inc St Petersburg, FL) and will be fitted with a bandoleer type antenna system, connected to a portable monitor that records core temp.

Doubly-labeled Water. On Day 0, a fasting baseline urine sample is collected, a metabolic weight taken and a DEXA scan performed. Following this, subjects will drink from a stock solution with 0.22g/kg total body water (TBW) of $H_2{}^{18}O$ (Cambridge Isotopes, Cambridge, MA) and 0.115 g/kg TBW of ^2H_2O (Isotec Inc., Miamisburg, OH or Cambridge Isotopes, Cambridge, MA), followed by a 100-200ml of tap water used to rinse the dose container. Following 2 voids, two consecutive urine samples are collected (at 4 and 6.5 hours after dosing). For the next 14 days after dosing urine samples are collected each morning after breakfast. On Day14, a metabolic weight and DEXA scan is repeated. Additional DLW measurements may be used to monitor compliance periodically throughout the study (at 1 month for Phase 1). Urine samples will be collected on days 0, 7 and 14 only for these additional measurements.
The ^2H and ^{18}O isotope elimination rates (k_H and k_O) will be calculated using linear regression using the isotopic enrichment relative to predose enrichment of the urine samples from the beginning (days 1 and 2 postdose) and end of the metabolic study (days 13 and 14). Total body water will be determined form the enrichment at time zero, obtained from the regression line. The rate of CO_2 production is calculated using the equations of Schoeller et al. [159] and later modified [160] as follows: rCO2 = $(N/2.078)(1.007k_O-1.041k_H)-0.0246rH_2O_f$, where N is the total body water and rH_2O_f is the rate of fractionated evaporative water loss which is estimated to be $1.05N\ (1.007k_O - 1.041k_H)$. Energy expenditure is calculated by multiplying rCO_2 by the energy equivalent of CO_2 for an assumed RQ of 0.882 (food quotient of the diet) and estimated changes in body energy stores as previously described [161]. We will also take into account the 24-h RQ measured a few days after in the respiratory chamber. Energy intake will also be estimated by subtracting energy expenditure from the change in body weight (kg) and body composition (DEXA) over the 14 day period according the methods of Schulz [162].

Exercise efficiency. Whole-body exercise efficiency will be determined using measures of O_2 consumption and CO_2 production (Vmax, Sensormedics, Yorba Linda, CA) obtained in the steady-state during the 3rd and 4th min of three workloads on a bicycle ergometer, i.e. 40, 80 and 120 watts at 60 rpm (or 30, 60 and 90 watts if necessary). Respiratory exchange will also be measured at baseline in resting conditions sitting on the bike and at freeload pedaling at 60 rpm. Delta efficiency will be calculated using the slope of the regression line between oxygen consumption and external workload [163].

Oxidative Stress

DNA Damage

Comet Assay for DNA damage – Single-cell gel electrophoresis (SCGE, comet assay) provides a very sensitive method for detecting strand breaks and measuring repair kinetics at the level of single cells [56]. A

variety of possible modifications to the assay facilitate the detection of single-strand breaks, incomplete excision repair sites, and interstrand cross-links. In addition to the above, DNA fragmentation associated with cell death or related to apoptosis can be evaluated with the comet assay. After initial treatment, cells are embedded in agarose layers, lysed, and electrophoresed. After staining with a fluorescent DNA binding dye, cells with increased DNA damage display increased DNA migration from the nucleus toward the anode, thus forming the shape of a "comet". The comet assay takes 2 days and utilizes a small number of cells. Briefly, lymphocytes are suspended in low melting point agarose and immobilized on slides. The slides are then treated with a gentle lysing solution, followed by a highly alkaline solution (>pH 12.6) to expose AP and other alkali labile sites. Alternatively, lysed cells can be treated with DNA glycosylases to reveal damaged DNA bases. The slides are then placed into an electrophoresis chamber. Following electrophoresis, the slides are fixed and stained. For quantification, digital imaging will be used. There is a commercially available kit from Trevigen for conducting the comet assay and the DNA repair proteins we propose to use are routinely purified in Dr. Deutsch laboratory.

Determination of baseless sites in DNA – The action of ROS destabilize DNA bases that result in the formation of baseless DNA (apurinic/apyrimidinic=AP). These baseless sites also arise as an intermediate in the repair of ROS damaged DNA bases such as 8-oxoguanine. A specific method for the detection of AP sites has been developed and is currently in use in Deutsch's laboratory. This method utilizes the reagent O-(carboxymethyl) hydroxylamine that has been reacted with biotin hydrazide in the presence of carbodimide. Aldehyde reactive probe (ARP) specifically reacts with AP sites in DNA, identifying them with biotin residues. The biotin-marked AP sites are determined using a color detection system in a slot-blot or ELISA type analysis and avidin/biotin complex conjugated to horseradish peroxidase. This system has been modified to use the more sensitive chemiluminescence detection kits (Boehringer-Mannheim) in a slot-blot apparatus. One AP site for every 10,000 bases of DNA (approximately 10fmol of AP sites) can be detected and requires as little as 300ng of DNA per reaction. Band densities are normalized with GAPDH expression.

Determination of 8-oxoguanine for DNA Damage - DNA isolated from lymphocytes will be used to determine the amount of 8-oxoG using gas chromatography-mass spectroscopy (GC-MS) with selected ion monitoring (SIM). Briefly, extracted DNA will be enzymatically hydrolyzed. Derivatization of the hydrolyzed DNA will be carried out using bis(trimethylsilyl)trifluoroacetamide (BSTFA) containing 1% trimethylchlorosilane and acetonitrile (1:1) in a total volume of 70 ml at 130°C for 20 min in vials flushed with nitrogen. Derivatized samples will then be injected into the GC-MS using SIM. Quantification of the modified DNA base will be carried out using specific ions eluting from the GC column. The stable isotope for 8-oxoG is already in use. Alternatively, urine samples could be evaluated for the presence of the free 8-oxoG base using GC-MS.

Isoprostanes. The isoprostane that has been most often studied (shown to be a potential marker for oxidative stress) is 8-epi PGF2 alpha, although there are several names for this compound. We will measure 8-epi PGF2 alpha as an isoprostane marker of oxidative stress [164] using HPLC/MS-MS. Briefly, an internal standard (deuterated 8-epi PGF2 alpha, Caymen Chemical) will be added to urine, followed by addition of 15% KOH and allowed to stand for 30 min. The sample is then subjected to solid phase extraction after adjusting pH. The sample is dried and resuspended in 20% acetonitrile in water and subjected to HPLC MS/MS. We have analyzed several isoprostanes, including 8-epi PGF2 alpha using our HPLC MS/MS (Finnigan TSQ 7000) and are currently piloting the method in smokers vs. non-smokers.

Carbonyl Content. Serum samples will be analyzed for carbonyl content, an indicator of oxidative damage to proteins. The method was described by Reznick and Packer [165]. Briefly, serum samples are combined with 2,4 dinitrylphenylhydrazine. The carbonyls in the sample react with the 2,4 DNP to form protein hydrazones. The product is measured spectrophotometrically at 355-390. Each sample is scanned against a sample treated with 2.5 M HCl. Carbonyl content is calculated from the absorption spectra using an absorption coefficient of 22,000 M^{-1} cm^{-1}.

CVD markers and vascular function

Plasma lipids and lipoproteins. Serum lipids will be analyzed using a Beckman-Coulter Synchron CX7 (Brea, CA). Serum cholesterol will be assayed by the cholesterol esterase/oxidase/peroxidase method. Triacylglycerols will be measured using the GPO-Trinder method. HDL-C will be measured following precipitation of apoB containing lipoproteins with 50,000 mol wt. dextran sulfate (Sigma Chemical Co.). LDL cholesterol is calculated using the Friedewald equation. The coefficient of variation for these assays is less than 2.0%. Our Clinical Laboratory participates in CDC's ongoing lipid standardization program.

Blood pressure measurements. Blood pressure measurements will be taken in a quiet room where no other activity is taking place and where temperature fluctuations are minimal. All measurements will be taken on the right arm while the volunteer is seated in the room and allowed to rest for 5 min. before the cuff is wrapped around the arm. Blood pressure will be taken twice by a nurse certified for blood pressure measurements.

Plasma hemostatic factors, inflammatory markers and homocysteine. Factor VII and fibrinogen will be assayed on an Instrumentation Laboratory ACL 3000+ (Lexington, MA). Factor VII activity is assayed by determining the ability of test plasma to correct the clotting time of factor VII-deficient plasma. Factor VII activity is expressed relative to serial dilution of pooled plasma. Fibrinogen will be measured following a standard protocol [166]. C-reactive protein, IL-6 and homocysteine will be measured by automated immunoassay with chemiluminescent detection on a DPC-Immulite instrument with reagents supplied by the instrument manufacturer.

Endothelial Function**.** Endothelial function will be estimated by high-resolution ultrasound measurements of changes in brachial artery diameter in response to reactive hyperemia following ischemia (brachial artery flow-mediated dilation) as described by Celemajer et al [167]. Prior to each measurement, subjects will rest for 10 min in a supine position. A baseline ultrasound image of the brachial artery will be obtained just proximal to the olecranon process of the elbow. The center of the vessel will be identified when the clearest images of the anterior and posterior walls of the artery are visualized. The transmit zone will be set to the levels of the anterior vessel wall and the depth and gain settings will be optimized to identify the lumen-vessel wall interface. All measurements will be performed with the non-dominant arm with the forearm extended and slightly supinated. Reactive hyperemia will be induced by inflation of a blood pressure cuff to 300-mm Hg for 5 min. The position of the cuff will be approximately 2-3cm distal to the olecranon process. Images of the brachial artery will be obtained 30 seconds prior to inflation (baseline), at the end of each minute during inflation, and continuously for 6 min after cuff deflation. All images will be recorded on super VHS videotape. Vessel diameters will be measured from recordings at 2 different locations relative to an anatomical marker and across 3 cardiac cycles during the diastolic phase.

Insulin Sensitivity and Secretion

To determine insulin sensitivity, a frequently sampled intravenous glucose tolerance test (FSIGT) with the addition of exogenous insulin injection will be performed [168]. Subjects will be studied after an

overnight fast while residing on the metabolic ward (Figure 2). After one hour of rest (RMR measure), 2 IV lines will be placed. Briefly, 300mg/kg glucose is injected at TIME 0, followed by collection of blood samples (4 cc) at 1, 3,4, 5, 6, 7, 8, 10, 12, 14, 16, and 19 min. At TIME=20 min., a bolus of insulin (0.03 U/kg body weight) is given, and frequent sampling resumes at 22, 32, 24, 25, 27, 30, 40, 50, 60, 60, 80, 100, 120, 140, 160 and 180 min. Each blood sample is analyzed for glucose and insulin. These data are then submitted for calculation of the insulin Sensitivity Index (S_I), glucose effectiveness (S_G), and AIRg using the Minimal Model method of Bergman [169]. The minimal model analysis is accomplished using a program developed for a PC (MINMOD-PC, © R. Bergman). Total blood collected during this procedure is less than 150 ml. To date, the clinical staff of the Pennington Center has performed over 250 determinations of insulin sensitivity in lean and obese individuals.

Neuroendocrine axes

Assessment of diurnal leptin rhythm will be performed using 24-h sampling. GH secretion will be assessed immediately before and during the sleep-induced rise in GH secretion as previously described [170]. Cortisol secretion (meal-related increases, and diurnal variation) will be assessed using salivary cortisol concentrations [171,172]. This method integrates the minute-to-minute fluctuations in plasma cortisol. The thyroid axis will be measured the following morning after stimulation with TRH [83]. The activity of the gonadal axis will be tested by measurement of SHBG, free testosterone and estradiol levels [170]. The experimental protocol for the neuroendocrine axes is shown in figure 3.

Figure 3

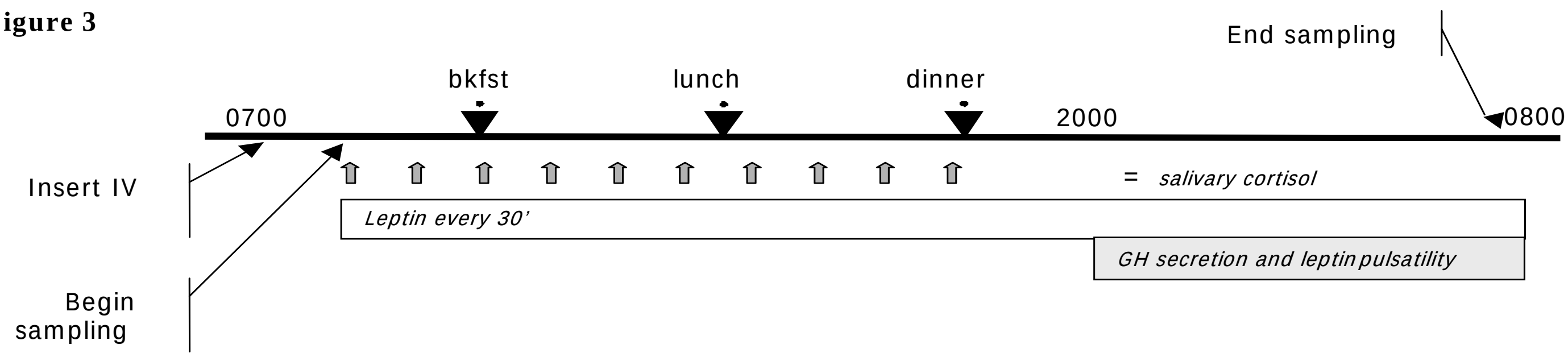

Autonomic activity

Spectral analysis of heart rate variability. Autonomic function will be assessed in the fasting state and 30-min. after the ingestion of a meal by spectral analysis of heart rate variability. Subjects will remain in a semi-supine position in a quiet room, while breathing at a controlled rate of 15 breaths/min. They will be instructed to breathe less deeply than normal in order to avoid hyperventilation. Heart rate signals will then be acquired continuously for 5 min. using an ECG lead II, Lifepack 9P (Physio-Control, Redmond,VA), samples at 500 Hz by analogue/digital converter (PCL-812 PC-LabCard, Omega Scientific Company, Stamford,CT). Subjects will then receive a standard meal (Ensure, Abbott Laboratories) containing 40% of RMR measured at baseline of the exercise efficiency test. Heart rate will be measured for the next 30 min. Data will be analyzed by spectral analysis as described by Gao et al [173]

24-h urinary norepinephrine excretion. 24-h urine will be collected while the subjects spend a day in energy balance in the respiratory chamber Norepinephrine excretion rate will be determined as an index of sympathetic nervous activity. It is reproducible in sedentary condition and predicts body weight changes in Pima Indian males [174].

Muscle/fat biopsy for gene expression

Fat biopsy. After cleansing the skin on the abdomen with povidone-iodine solution, and placing a sterile drape, topical anesthesia is administered with 5mL of a 50% -50% mixture of lidocaine and bupivacaine. A 0.75-cm incision is made in the skin and a 4-6mm Bergstrom needle inserted to collect under aspiration approximately 250-350mg of adipose tissue. The sample is washed in sterile PBS and 75% of it snap frozen in liquid nitrogen, the remaining placed in osmium tetraoxide (potential fat cell morphology). Upon completion of the biopsy, the incision is closed with a sterile bandage, and antibiotic ointment / sterile dressing applied.

Muscle biopsy**.** A Vastus Lateralis muscle biopsy will be performed using the technique of Bergstrom. After cleansing the skin with povidone-iodine solution, the skin, adipose tissue and skeletal muscle fascia are anesthetized using 5mL of a 50%/50% mixture of bupivicaine and lidocaine (final concentrations 1.0% and 0.125%). The skin is incised (0.75cm) with a #11 scalpel. The fascia fibers are separated with the blunt edge of the scalpel and the Bergstrom needle (4mm) inserted into the vastus lateralis. After suction is applied, approximately 50mg of tissue is cut and removed. Two or 3 passes will be used to obtain at least 200mg of muscle. Pressure is applied and the skin is closed with sterile tape. After cleaning the sample, muscle will be snap frozen in liquid nitrogen. Dr. Smith has performed hundreds of these biopsies.

Total RNA extraction and gene expression**.** RNA will be extracted from individual muscle and subcutaneous fat biopsies containing about 100 mg of tissue, giving yields of 10-20µg total RNA. 50-100mg tissue sample is homogenized in 1mL Tri-Reagent using a glass-Teflon homogenizer. Homogenates are stored for 5 min. at room temperature to permit the complete dissociation of nucleoprotein complexes. RNA is then precipitated, washed and solubilized according to standard techniques.

Two complimentary strategies will be used. *First*, we will use a candidate gene approach, i.e. selecting known genes and measuring gene expression by real-time quantitative fluorescent RT-PCR using the Taqman™ method. The 49 selected genes are listed in an Appendix. *Second*, expression profiling, will be performed using cDNA microarrays [175-177]. This technique allows for simultaneous measurement of thousands of mRNAs by hybridizing small amounts of RNA to multiple DNA sequences spotted onto glass slides. We have purchased an oligo library including more than 19,000 genes.

Macronutrient Self-Selection and Fat Preference

Dr. Geiselman has developed a macronutrient self-selection paradigm (MSSP©) designed to vary fat content significantly and systematically with sugar, complex carbohydrate, and protein content [107]. We have also developed a food preference questionnaire (FPQ©) based on the same design. However, specific foods listed on the FPQ© and those presented for selection/consumption in the MSSP© are mutually exclusive. All foods used in the MSSP© are commercial foods available from the supermarket shelves, and most of the foods in this paradigm are often eaten as snacks. Foods included in the MSSP© require little or no preparation. Other foods that are common sources of fat in the American diet [178] but that require considerable preparation are included on the FPQ©. These instruments have strong test-retest reliability and have been validated with respect to long-term macronutrient intake in men [107] and in women [179]. They include foods that are the top sources of fat for Caucasian and minorities in the Delta regions of Louisiana [180].

In the proposed study, the MSSP© will be administered at baseline only. The MSSP© and FPQ© questionnaire will be administered before subjects are put on the AHA diet. The FPQ© will then be administered at 3 and 6 mos. We will test the following hypotheses: 1) Greater preference for and intake of high-fat/high-sugar foods, will be associated with greater difficulty in maintaining CR; 2) Decreasing

fat preference scores from baseline is a behavioral adaptation associated with greater success in maintaining CR.

Power Calculation and Data Analysis

The power calculations and sample size determination were done for the primary endpoint, 24-h energy expenditure (24EE). It is assumed that during the intervention and at the end of the two-year study the change from baseline will be analyzed and the effect of two different types of intervention (CR and CR+PA) will be tested. Since there are very few long-term longitudinal data from chamber studies available for the estimate of a within subject variability, we took a conservative approach assuming the coefficient of variation (CV) over a 2-year period to be 5%. Indeed, the inter-individual CV is 2.4% for the chamber at NIH [181] and 4.9% at the Pennington Center. In subjects similar to those targeted in this study with a follow-up >2 yr., the intra-individual CV was 5.3% (Tataranni, NIH data). A minimal variability of means is assumed and the detectable differences are 5, 10, 15 and 20% from baseline [182]. The significance level is set to $\alpha=0.05$ and the power is required to be at least 80%. Additional power calculations were done for adjusted 24-h EE. The estimate of the within subject coefficient of variation resulted from the regression analysis of 24EE on fat-free mass, fat mass, age and sex. For the summarized results on sample sizes, see Table 2, below. As can be seen, assuming a 5% within subject CV (conservative), only 11 subjects in each group need to complete the study for picking a 10% decrease in sedentary energy expenditure measured in our respiratory chambers. This number of subjects allows us to detect a 15% decrease in total daily energy expenditure by doubly labeled water, assuming a 7.5% within subject variability. One therefore can feel confident that by enrolling 30 subjects per group, one will have a sufficient number of subjects to detect these rather small differences in energy metabolism**.**

In the final analysis we will investigate the change from baseline for all continuous variables. The analysis will be carried out in two different ways. Using an ANOVA method with treatment groups as fixed effect, we will do a simple analysis of end-of-study change from baseline. The second one is the more detailed and precise analysis using a mixed model approach considering a repeated measures design method with treatment groups and time as fixed effects and subject as random effect. The covariance matrix of the multivariate response for each subject will be modeled appropriately and estimated using the maximum likelihood method. The drop out rates will be compared across all treatment groups (3) testing for equality of proportions using the χ^2 test. For all analyses, SAS Version 8.1 (or higher) will be used.

**TABLE 2** Within subject CV **(%)**	Detectable difference (% from baseline)	n/group	Total N at the end of the study	Power (%)
Chamber method				
3	5	15	45	80
3	10	5	15	84
3	15	3	9	85
3	20	3	9	98
5	5	40	120	80
5	10	11	33	80
5	15	6	18	85
5	20	4	12	86
Doubly labeled water method				
7.5	10	23	69	80
7.5	15	11	33	81
7.5	20	7	21	83

RISK / BENEFIT ASSESSMENT

Potential Risk and Discomforts
The study involves the risk associated with small doses of radiation equivalent to 12-hr. exposure to outdoor radiation (DEXA) and to a chest X-ray series (CT scan). The fat and muscle biopsies carry the risk of local infections and small scars. The IVGTT and the neuroendocrine test carry the risk of bruises at the IV site.

Risk Classification
The risks associated with the proposed procedures are less than the potential benefits to be gained by the volunteers.

Minimizing Risks/Confidentiality
While there are some potential risks to the proposed study, the design makes every attempt to prevent the possibility of an adverse event. Regarding the interventions, efforts to minimize the potential risks of the assessment methods and outcome variables include frequent monitoring by physicians and investigators to assure that no volunteer suffers any adverse effects from participating in the research.
All volunteers are assured of their anonymity and confidentiality both verbally and in the informed consent form. The clinical facilities are strictly limited to the staff of the research institution and to research volunteers. This is accomplished by a variety of stringent security measures. All medical records are stored in locked areas. Access to these areas is limited to the clinical support staff, director of the clinical facilities, and the PIs. Volunteers' medical records are filed according to ID numbers. All forms on the chart, with the exception of consent form, display only the ID number. Electronic data storage is similarly restricted with only the PIs and authorized persons having access to databases containing confidential clinical records, i.e. those containing name, Social Security Number or other identifying information

Potential Benefits
All volunteers will gain information about their current health, and will also receive at no charge diet and exercise advice. Most of them (intervention) will loose weight and become normal weight. Those in the control group will benefit of the education to eat a healthy low fat diet

Risk / Benefit Ratio
There is no unreasonable risk in this study.

Payment for Participation
Payment for time and inconvenience will be as follows: $10.00/day in the study from enrollment and $1000.00 per block of fully completed testing.

Financial Obligations of the Subjects
The volunteer has no financial obligation relating to this study.

G. LITERATURE CITED

1. McCay, C. M., Crowel, M. F. & Maynard, L. A. The effect of retarded growth upon the length of the life span and upon th eultimate body size. *J Nutr* **10**, 63-79 (1935).
2. Barrows, C. H. & Kokkonen, G. C. in *Nutritional approaches to aging research.* (ed. Moment, G. B.) (CRC Press, Inc., Boca Raton, FL, 1982).
3. Weindruch, R. & Walford, R. L. *The retardation of aging and disease by dietary restriction.* (Charles C. Thomas Publisher, Springfield, IL, 1988).
4. Kemnitz, J. W. et al. Dietary restriction of adult male rhesus monkeys: design, methodology, and preliminary findings from the first year of study. *J Gerontol* **48**, B17-26. (1993).
5. Bodkin, N. L., Ortmeyer, H. K. & Hansen, B. C. Long-term dietary restriction in older-aged rhesus monkeys: effects on insulin resistance. *J Gerontol A Biol Sci Med Sci* **50**, B142-7. (1995).
6. Lane, M. A., Black, A., Ingram, D. K. & Roth, G. S. Calorie restriction in non-human primates: implications for age-related disease risk. *J Anti-Aging Med* **1**, 315-326 (1998).
7. Hansen, B. C. in *FASEB* 400 (Orlando, FL, 2001).
8. (National Academy Press, Washington, DC, 1991).
9. Schneider, E. L., Vining, E. M., Hadley, E. C. & Farnham, S. A. Recommended dietary allowances and the health of the elderly. *N Engl J Med* **314**, 157-60. (1986).
10. Eveleth, P. B. & Tanner, J. M. *Worldwide Variation in Human Growth* (Cambridge University Press, New York, 1990).
11. Ellison, P. T. Developmental influences on adult ovarian function. *Am J Human Biol* **8**, 725-734 (1996).
12. Ellison, P. T. in *Variability in Human Fertility: A Biological Anthropological Approach.* (eds. Rosetta, L. & Mascie-Taylor, N. C. G.) 69-90 (Cambridge University Press, New York, 1996).
13. Prentice, A. M., Lunn, P. G., Watkinson, M. & Whitehead, R. G. Dietary supplementation of lactating Gambian women. II. Effect on maternal health, nutritional status and biochemistry. *Hum Nutr Clin Nutr* **37**, 65-74. (1983).
14. Ellison, P. T., Panter-Brick, C., Lipson, S. F. & O'Rourke, M. T. The ecological context of human ovarian function. *Hum Reprod* **8**, 2248-58. (1993).
15. Lager, C. & Ellison, P. T. Effect of moderate weight loss on ovarian function assessed by salivary progesterone measurements. *Am J Human Biol* **2**, 303-312 (1990).
16. Roberts, S. B., Paul, A. A., Cole, T. J. & Whitehead, R. G. Seasonal changes in activity, birth weight and lactational performance in rural Gambian women. *Trans R Soc Trop Med Hyg* **76**, 668-78 (1982).
17. Lee, R. B. *The !Kung San: Men, Women, and Work in a Foraging Society.* (University of Chicago Press, Chicago, IL, 1979).
18. Martorell, R. in *Social and Biological Predictors of Nutritional Status, Physical Growth, and Neurological Development.* (ed. Greene, L. S., Johnston, F.E.) 81-106 (Academic Press, New York, 1980).
19. Ulijaszek, S. J. in *Diet and Disease.* (eds. Harrison, G. A. & Waterlow, J. C.) 137-154 (Cambridge University Press, New York, 1990).
20. Keys, A., Brozek, J., Henschel, A., Mickelson, O. & Taylor, H. *The Biology of Human Starvation.* (University of Minnesota Press, Minneapolis, MN, 1950).
21. Kagawa, Y. Impact of Westernization on the nutrition of Japanese: changes in physique, cancer, longevity and centenarians. *Prev Med* **7**, 205-17. (1978).
22. Vallejo, E. A. La dieta de hambre a dias alternos in la alimentacion de los viejos [in Spanish]. *Rev Clin Exp* **63**, 25-26 (1957).

23. Stunkard, A. J. Nutrition, aging and obesity. pp. 253-84. *In: Rockstein M, Sussman ML, ed. Nutrition, longevity, and aging. New York, Academic Press* **104**, 30 (1976).
24. Bathalon, G. P. et al. Metabolic, Psychological, and Health Correlates of Dietary Restraint in Healthy Postmenopausal Women. *J Gerontol A Biol Sci Med Sci* **56**, M206-M211. (2001).
25. Fricker, J., Rozen, R., Melchior, J. C. & Apfelbaum, M. Energy-metabolism adaptation in obese adults on a very-low-calorie diet. *Am J Clin Nutr* **53**, 826-30. (1991).
26. Vansant, G., Van Gaal, L., Van Acker, K. & De Leeuw, I. Short and long term effects of a very low calorie diet on resting metabolic rate and body composition. *Int J Obes* **13**, 87-9. (1989).
27. Keesey, R. E. Physiological regulation of body weight and the issue of obesity. *Med Clin North Am* **73**, 15-27. (1989).
28. Astrup, A. et al. Meta-analysis of resting metabolic rate in formerly obese subjects. *Am J Clin Nutr* **69**, 1117-22. (1999).
29. Leibel, R. L., Rosenbaum, M. & Hirsch, J. Changes in energy expenditure resulting from altered body weight. *N Engl J Med* **332**, 621-8. (1995).
30. Weigle, D. S., Sande, K. J., Iverius, P. H., Monsen, E. R. & Brunzell, J. D. Weight loss leads to a marked decrease in nonresting energy expenditure in ambulatory human subjects. *Metabolism* **37**, 930-6. (1988).
31. Astrup, A., Buemann, B., Toubro, S., Ranneries, C. & Raben, A. Low resting metabolic rate in subjects predisposed to obesity: a role for thyroid status. *Am J Clin Nutr* **63**, 879-83. (1996).
32. Weigle, D. S. & Brunzell, J. D. Assessment of energy expenditure in ambulatory reduced-obese subjects by the techniques of weight stabilization and exogenous weight replacement. *Int J Obes* **14**, 69-77;discussion 77-81. (1990).
33. Weyer, C. et al. Energy metabolism after 2 y of energy restriction: the biosphere 2 experiment. *Am J Clin Nutr* **72**, 946-53. (2000).
34. Jenkins, R. R. & Goldfarb, A. Introduction: oxidant stress, aging, and exercise. *Med Sci Sports Exerc* **25**, 210-2. (1993).
35. Rising, R., Keys, A., Ravussin, E. & Bogardus, C. Concomitant interindividual variation in body temperature and metabolic rate. *Am J Physiol* **263**, E730-4. (1992).
36. Sohal, R. S. & Allen, R. G. Relationship between metabolic rate, free radicals, differentiation and aging: a unified theory. *Basic Life Sci* **35**, 75-104 (1985).
37. Orr, W. C. & Sohal, R. S. Extension of life-span by overexpression of superoxide dismutase and catalase in Drosophila melanogaster. *Science* **263**, 1128-30. (1994).
38. Melov, S. et al. Extension of life-span with superoxide dismutase/catalase mimetics. *Science* **289**, 1567-9. (2000).
39. Sohal, R. S. & Weindruch, R. Oxidative stress, caloric restriction, and aging. *Science* **273**, 59-63. (1996).
40. Harman, D. Aging: a theory based on free radical and radiation chemistry. *J Gerontol* **11**, 298-300 (1956).
41. Stadtman, E. R. Protein oxidation and aging. *Science* **257**, 1220-4. (1992).
42. Terman, A. & Brunk, U. T. Lipofuscin: mechanisms of formation and increase with age. *Apmis* **106**, 265-76. (1998).
43. Pratico, D. et al. IPF2alpha-I: an index of lipid peroxidation in humans. *Proc Natl Acad Sci U S A* **95**, 3449-54. (1998).
44. Bohr, V. A. & Dianov, G. L. Oxidative DNA damage processing in nuclear and mitochondrial DNA. *Biochimie* **81**, 155-60. (1999).
45. Adelman, R., Saul, R. L. & Ames, B. N. Oxidative damage to DNA: relation to species metabolic rate and life span. *Proc Natl Acad Sci U S A* **85**, 2706-8. (1988).

46. Weeda, G. et al. A presumed DNA helicase encoded by ERCC-3 is involved in the human repair disorders xeroderma pigmentosum and Cockayne's syndrome. *Cell* **62**, 777-91. (1990).
47. Troelstra, C. et al. ERCC6, a member of a subfamily of putative helicases, is involved in Cockayne's syndrome and preferential repair of active genes. *Cell* **71**, 939-53. (1992).
48. Henning, K. A. et al. The Cockayne syndrome group A gene encodes a WD-repeat protein which interacts with CSB protein and a subunit of RNA Pol II transcription factor IIH (TFIIH). *Cell* **82**, 555-564 (1995).
49. Savitsky, K. et al. A single ataxia telangiectasia gene with a product similar to PI-3 kinase. *Science* **268**, 1749-53. (1995).
50. Dizdaroglu, M., Nackerdien, Z., Chao, B. C., Gajewski, E. & Rao, G. Chemical nature of in vivo DNA base damage in hydrogen peroxide-treated mammalian cells. *Arch Biochem Biophys* **285**, 388-90. (1991).
51. Cabrera, M., Nghiem, Y. & Miller, J. H. mutM, a second mutator locus in Escherichia coli that generates G.C---- T.A transversions. *J Bacteriol* **170**, 5405-7. (1988).
52. Shibutani, S., Takeshita, M. & Grollman, A. P. Insertion of specific bases during DNA synthesis past the oxidation- damaged base 8-oxodG. *Nature* **349**, 431-4. (1991).
53. Loeb, L. A. & Preston, B. D. Mutagenesis by apurinic/apyrimidinic sites. *Annu Rev Genet* **20**, 201-30 (1986).
54. Sagher, D. & Strauss, B. Insertion of nucleotides opposite apurinic/apyrimidinic sites in deoxyribonucleic acid during in vitro synthesis: uniqueness of adenine nucleotides. *Biochemistry* **22**, 4518-26. (1983).
55. Fraga, C. G., Shigenaga, M. K., Park, J. W., Degan, P. & Ames, B. N. Oxidative damage to DNA during aging: 8-hydroxy-2'-deoxyguanosine in rat organ DNA and urine. *Proc Natl Acad Sci U S A* **87**, 4533-7. (1990).
56. Atamna, H., Cheung, I. & Ames, B. N. A method for detecting abasic sites in living cells: age-dependent changes in base excision repair. *Proc Natl Acad Sci U S A* **97**, 686-91. (2000).
57. Ross, R. Atherosclerosis--an inflammatory disease. *N Engl J Med* **340**, 115-26. (1999).
58. Ridker, P. M., Glynn, R. J. & Hennekens, C. H. C-reactive protein adds to the predictive value of total and HDL cholesterol in determining risk of first myocardial infarction. *Circulation* **97**, 2007-11. (1998).
59. Ridker, P. M., Buring, J. E., Shih, J., Matias, M. & Hennekens, C. H. Prospective study of C-reactive protein and the risk of future cardiovascular events among apparently healthy women. *Circulation* **98**, 731-3. (1998).
60. Koenig, W. et al. C-Reactive protein, a sensitive marker of inflammation, predicts future risk of coronary heart disease in initially healthy middle-aged men: results from the MONICA (Monitoring Trends and Determinants in Cardiovascular Disease) Augsburg Cohort Study, 1984 to 1992. *Circulation* **99**, 237-42. (1999).
61. Libby, P. & Ridker, P. M. Novel inflammatory markers of coronary risk: theory versus practice. *Circulation* **100**, 1148-50. (1999).
62. Apfelbaum, M. Adaptation to changes in caloric intake. *Prog Food Nutr Sci* **2**, 543-59 (1978).
63. Melby, C. L., Sylliaasen, S. & Rhodes, T. Diet-induced weight loss and metabolic changes in obese women with high versus low prior weight loss/regain. *Nutr Res* **11**, 971-978 (1991).
64. Shetty, P. S. & Kurpad, A. V. Role of the sympathetic nervous system in adaptation to seasonal energy deficiency. *Eur J Clin Nutr* **44**, 47-53. (1990).
65. Landsberg, L. & Young, J. B. in *Nutritional factors: modulating effects on metabolic processes* (eds. Beers, R. F. & Bassett, E. G.) 155-174 (Raven Press, New York, 1981).
66. Landsberg, L. & Young, J. B. The role of the sympathetic nervous system and catecholamines in the regulation of energy metabolism. *Am J Clin Nutr* **38**, 1018-24. (1983).

67. Velthuis-te Wierik, E. J., van den Berg, H., Schaafsma, G., Hendriks, H. F. & Brouwer, A. Energy restriction, a useful intervention to retard human ageing? Results of a feasibility study. *Eur J Clin Nutr* **48**, 138-48. (1994).
68. Wood, P. D., Stefanick, M. L., Williams, P. T. & Haskell, W. L. The effects on plasma lipoproteins of a prudent weight-reducing diet, with or without exercise, in overweight men and women. *N Engl J Med* **325**, 461-6. (1991).
69. Dattilo, A. M. & Kris-Etherton, P. M. Effects of weight reduction on blood lipids and lipoproteins: a meta- analysis. *Am J Clin Nutr* **56**, 320-8. (1992).
70. Perticone, F. et al. Obesity and body fat distribution induce endothelial dysfunction by oxidative stress: protective effect of vitamin C. *Diabetes* **50**, 159-65. (2001).
71. Ting, H. H. et al. Vitamin C improves endothelium-dependent vasodilation in forearm resistance vessels of humans with hypercholesterolemia. *Circulation* **95**, 2617-22. (1997).
72. Heitzer, T., Just, H. & Munzel, T. Antioxidant vitamin C improves endothelial dysfunction in chronic smokers. *Circulation* **94**, 6-9. (1996).
73. Taddei, S., Virdis, A., Ghiadoni, L., Magagna, A. & Salvetti, A. Vitamin C improves endothelium-dependent vasodilation by restoring nitric oxide activity in essential hypertension. *Circulation* **97**, 2222-9. (1998).
74. Kelley, D. E. Effects of weight loss on glucose homeostasis in NIDDM. *Diabetes Reviews* **3**, 366-377 (1995).
75. Ryan, A. S. Insulin resistance with aging: effects of diet and exercise. *Sports Med* **30**, 327-46. (2000).
76. Ross, R. et al. Reduction in obesity and related comorbid conditions after diet-induced weight loss or exercise-induced weight loss in men. A randomized, controlled trial. *Ann Intern Med* **133**, 92-103. (2000).
77. Gazdag, A. C., Sullivan, S., Kemnitz, J. W. & Cartee, G. D. Effect of long-term caloric restriction on GLUT4, phosphatidylinositol- 3 kinase p85 subunit, and insulin receptor substrate-1 protein levels in rhesus monkey skeletal muscle. *J Gerontol A Biol Sci Med Sci* **55**, B44-6; discussion B47-8. (2000).
78. Cefalu, W. T. et al. Influence of caloric restriction on the development of atherosclerosis in nonhuman primates: progress to date. *Toxicol Sci* **52**, 49-55. (1999).
79. Walford, R. L., Mock, D., MacCallum, T. & Laseter, J. L. Physiologic changes in humans subjected to severe, selective calorie restriction for two years in biosphere 2: health, aging, and toxicological perspectives. *Toxicol Sci* **52**, 61-5. (1999).
80. Walford, R. L., Weber, L. & Panov, S. Caloric restriction and aging as viewed from Biosphere 2. *Receptor* **5**, 29-33. (1995).
81. Shulman, G. I. Cellular mechanisms of insulin resistance. *J Clin Invest* **106**, 171-6 (2000).
82. Shimokawa, I. & Higami, Y. Leptin and anti-aging action of caloric restriction. *J Nutr Health Aging* **5**, 43-8 (2001).
83. Roti, E., Minelli, R. & Salvi, M. Thyroid hormone metabolism in obesity. *Int J Obes Relat Metab Disord* **24 Suppl 2**, S113-5. (2000).
84. Fichter, M. M., Pirke, K. M. & Holsboer, F. Weight loss causes neuroendocrine disturbances: experimental study in healthy starving subjects. *Psychiatry Res* **17**, 61-72. (1986).
85. Everitt, A. V., Seedsman, N. J. & Jones, F. The effects of hypophysectomy and continuous food restriction, begun at ages 70 and 400 days, on collagen aging, proteinuria, incidence of pathology and longevity in the male rat. *Mech Ageing Dev* **12**, 161-72. (1980).
86. Meites, J. Evidence that underfeeding acts via the neuroendocrine system to influence aging processes. *Prog Clin Biol Res* **287**, 169-80 (1989).

87. Masoro, E. J. Food restriction in rodents: an evaluation of its role in the study of aging. *J Gerontol* **43**, B59-64. (1988).
88. Nelson, J. F. in *Modulation of Aging Processes by Dietary Restriction* (ed. Yu, B. P.) 37-55 (CRC Press, Boca Raton, FL, 1994).
89. Klebanov, S., Diais, S., Stavinoha, W. B., Suh, Y. & Nelson, J. F. Hyperadrenocorticism, attenuated inflammation, and the life-prolonging action of food restriction in mice. *J Gerontol A Biol Sci Med Sci* **50**, B79-82. (1995).
90. Armario, A., Montero, J. L. & Jolin, T. Chronic food restriction and the circadian rhythms of pituitary-adrenal hormones, growth hormone and thyroid-stimulating hormone. *Ann Nutr Metab* **31**, 81-7 (1987).
91. Herlihy, J. T., Stacy, C. & Bertrand, H. A. Long-term food restriction depresses serum thyroid hormone concentrations in the rat. *Mech Ageing Dev* **53**, 9-16. (1990).
92. Sabatino, F., Masoro, E. J., McMahan, C. A. & Kuhn, R. W. Assessment of the role of the glucocorticoid system in aging processes and in the action of food restriction. *J Gerontol* **46**, B171-9. (1991).
93. Sonntag, W. E. et al. Pleiotropic effects of growth hormone and insulin-like growth factor (IGF)-1 on biological aging: inferences from moderate caloric- restricted animals. *J Gerontol A Biol Sci Med Sci* **54**, B521-38. (1999).
94. Ahima, R. S. et al. Role of leptin in the neuroendocrine response to fasting. *Nature* **382**, 250-2. (1996).
95. Kirkwood, T. B. Evolution of ageing. *Nature* **270**, 301-4. (1977).
96. Laughlin, G. A. & Yen, S. S. Hypoleptinemia in women athletes: absence of a diurnal rhythm with amenorrhea. *J Clin Endocrinol Metab* **82**, 318-21. (1997).
97. Mantzoros, C. S. Role of leptin in reproduction. *Ann N Y Acad Sci* **900**, 174-83 (2000).
98. Smith, S. R. in *Endocrinology and Metabolism Clinics of North America* (ed. Bray, G.) 921-942 (W.B Saunders, 1996).
99. Frystyk, J., Vestbo, E., Skjaerbaek, C., Mogensen, C. E. & Orskov, H. Free insulin-like growth factors in human obesity. *Metabolism* **44**, 37-44. (1995).
100. Nam, S. Y. et al. Effect of obesity on total and free insulin-like growth factor (IGF)-1, and their relationship to IGF-binding protein (BP)-1, IGFBP-2, IGFBP-3, insulin, and growth hormone. *Int J Obes Relat Metab Disord* **21**, 355-9. (1997).
101. Lee, E. J. et al. Acipimox potentiates growth hormone response to growth hormone- releasing hormone by decreasing serum free fatty acid levels in hyperthyroidism. *Metabolism* **44**, 1509-12. (1995).
102. Lee, C. K., Klopp, R. G., Weindruch, R. & Prolla, T. A. Gene expression profile of aging and its retardation by caloric restriction. *Science* **285**, 1390-3. (1999).
103. Lee, C. K., Weindruch, R. & Prolla, T. A. Gene-expression profile of the ageing brain in mice. *Nat Genet* **25**, 294-7. (2000).
104. Welle, S., Brooks, A. & Thornton, C. A. Senescence-related changes in gene expression in muscle: similarities and differences between mice and men. *Physiol Genomics* **5**, 67-73. (2001).
105. Green, S. M. & Blundell, J. E. Subjective and objective indices of the satiating effect of foods. Can people predict how filling a food will be? *Eur J Clin Nutr* **50**, 798-806. (1996).
106. Rolls, B. J., Hetherington, M. & Burley, V. J. The specificity of satiety: the influence of foods of different macronutrient content on the development of satiety. *Physiol Behav* **43**, 145-53 (1988).
107. Geiselman, P. J. et al. Reliability and validity of a macronutrient self-selection paradigm and a food preference questionnaire. *Physiol Behav* **63**, 919-28. (1998).
108. Geiselman, P. J. et al. Macronutrient self-selection in lean, obese and post-obese men and women following exercise. (Manuscript in preparation, 2001).

109. USDHHS. (United States Department of Health and Human Services, Centers for Disease Control and Prevention, National Center for Chronic Disease Prevention and Health Promotion, Atlanta, GA, 1996).
110. Blair, S. N. et al. Physical fitness and all-cause mortality. A prospective study of healthy men and women. *Jama* **262**, 2395-401. (1989).
111. Villeneuve, P. J., Morrison, H. I., Craig, C. L. & Schaubel, D. E. Physical activity, physical fitness, and risk of dying. *Epidemiology* **9**, 626-31. (1998).
112. Andersen, L. B., Schnohr, P., Schroll, M. & Hein, H. O. All-cause mortality associated with physical activity during leisure time, work, sports, and cycling to work. *Arch Intern Med* **160**, 1621-8. (2000).
113. First, M. B., Gibbon, M., Spitzer, R. L. & Williams, J. B. W. *User's guide for the structural clinical interview for DSM-IV Axis I Disorders (SCID-1, Version 2.0)* (American Psychiatric Press, Washington, DC, 1995).
114. *American Psyciatric Association. Diagnostic and Statistical Manual of Mental Disorders* (Author, Washington, DC, 1994).
115. Kutlesic, V., Williamson, D. A., Gleaves, D. H., Barbin, J. M. & Murphy-Eberenz, K. P. The interview for the diagnosis of eating disorders IV: Application to DSM-IV diagnostic criteria. *Psychological Assessment* **10**, 41-48 (1998).
116. Krauss, R. M. et al. Aha scientific statement: aha dietary guidelines: revision 2000: a statement for healthcare professionals from the nutrition committee of the american heart association. *J Nutr* **131**, 132-46. (2001).
117. Schofield, W. N. Predicting basal metabolic rate, new standards and review of previous work. *Hum Nutr Clin Nutr* **39**, 5-41. (1985).
118. Sours, H. E. et al. Sudden death associated with very low calorie weight reduction regimens. *Am J Clin Nutr* **34**, 453-61. (1981).
119. Benedict, F. G. *A study of prolonged fasting* (Carnegie Institute, Washington, DC, 1915).
120. Drenick, E. J. in *Obesity in Perspective* (ed. Bray, G. A.) (U.S. Government Printing Office, Washington, DC, 1975).
121. Pringle, T. H., Scobie, I. N., Murray, R. G., Kesson, C. M. & Maccuish, A. C. Prolongation of the QT interval during therapeutic starvation: a substrate for malignant arrhythmias. *Int J Obes* **7**, 253-61 (1983).
122. Greenway, F. L., Raum, W. J. & Atkinson, R. L. Higher caloric content preserves myocardial electrical activity during very-low-calorie dieting. *Obes Res* **2**, 95-99 (1994).
123. Axelrad, M. A. & Alexander, J. K. The electrocardiogram and cardiac anatomy in obesity. *Clinical Research* **13**, 25 (1965).
124. Weinsier, R. L. & Ullmann, D. O. Gallstone formation and weight loss. *Obes Res* **1**, 51-55 (1993).
125. Stone, B. G., Ansel, H. J., Peterson, F. J. & Gebhard, R. L. Gallbladder emptying stimuli in obese and normal-weight subjects. *Hepatology* **15**, 795-8. (1992).
126. American College of Sports Medicine Position Stand. The recommended quantity and quality of exercise for developing and maintaining cardiorespiratory and muscular fitness, and flexibility in healthy adults. *Med Sci Sports Exerc* **30**, 975-91. (1998).
127. Pollack, M. L. & al., e. Effects of frequency and duration of training on attrition and incidence of injury. *Med Sci Sports Exerc* **9**, 31 (1977).
128. Alpert, S. S. Optimal time response of the human body to food shortage and the two reservoir model. *Angewandte Systemanalyse Verlag TUV Rheinland Köln* **3**, 79-84 (1982).
129. Williamson, D. A. *Assessment of Eating Disorders: Obesity, anorexia, and bulimia nervosa* (Pergamon Press, New York, 1990).

130. Anonymous. Dieting and the development of eating disorders in overweight and obese adults. *Arch Intern Med* **160**, 2581-9. (2000).
131. Cooper, P. J., Taylor, M. J., Cooper, Z. & Fairburn, C. G. The development and validation of the Body Shape Questionnaire. *Int J Eat Disord* **6**, 485-494 (1987).
132. Womble, L. G., Williamson, D. A., Netemeyer, S. B. & Netemeyer, R. G. Risk factors for the development of bulimic symptoms in high school girls: A one-year longitudinal study. *J Gender, Culture and Health* **3**, 227-241 (1999).
133. Anderson, D. A., Williamson, D. A., Duchmann, E. G., Gleaves, D. H. & Barbin, J. M. Development and validation of a multifactorial treatment outcome measure for eating disorders. *Assessment* **6**, 7-20. (1999).
134. Diener, Emmons, Larsen & Griffin. The satisfaction with life scale. *Journal of Personality Assessment* **49**, 71-75 (1985).
135. Alfonso, V. C. in *Handbook of Assessment Measures for Eating Behaviors and Weight-related Problems* (ed. Allison, D.) 23-80 (Sage Publications, Thousand Oaks, CA, 1995).
136. Ware, J. E., Jr. & Sherbourne, C. D. The MOS 36-item short-form health survey (SF-36). I. Conceptual framework and item selection. *Med Care* **30**, 473-83. (1992).
137. Goldberg, D. P. & Hillier, V. F. A scaled version of the General Health Questionnaire. *Psychol Med* **9**, 139-45. (1979).
138. Beck, A. T., Brown, G. K. & Steer, R. A. *Beck Depression Inventory-II* (Psychological Corporation, San Antonio, TX, 1996).
139. Derogatis, L. R. *Symptom Checklist-90 manual* (Johns Hopkins University Press, Baltimore, 1977).
140. Peterson, M. J., Eger, C. E. & Brown, G. G. Meaningfulness, phonemic similarity, and sensory memory. *Journal of Experimental Psychology* **98**, 64-69 (1973).
141. Boone, K. B., Ponton, M. O., Gorsuch, R. L., Gonzales, J. J. & Miller, B. L. Factor analysis of four measures of prefrontal lobe functioning. *Archives of Clinical Neuropsychology* **13**, 585-595 (1998).
142. Conners, C. K. The computerized continuous performance test. *Psychopharmacol Bull* **21**, 891-2 (1985).
143. Schmidt, M. *The Rey Auditory Verbal Learning Test: A handbook* (Western Psychological Services, Los Angelas, CA, 1996).
144. Benton, A. L. *Revised Visual Retention Test* (The Psychological Corporation, New York, 1974).
145. Heymsfield, S. B. (Personal Communication, Manuscript to be Submitted).
146. Caro, J. F., Dohm, L. G., Pories, W. J. & Sinha, M. K. Cellular alterations in liver, skeletal muscle, and adipose tissue responsible for insulin resistance in obesity and type II diabetes. *Diabetes Metab Rev* **5**, 665-89 (1989).
147. Clausen, J. O. et al. Insulin sensitivity index, acute insulin response, and glucose effectiveness in a population-based sample of 380 young healthy Caucasians. Analysis of the impact of gender, body fat, physical fitness, and life-style factors. *J Clin Invest* **98**, 1195-209 (1996).
148. Kissebah, A. H. & Peiris, A. N. Biology of regional body fat distribution: relationship to non-insulin- dependent diabetes mellitus. *Diabetes Metab Rev* **5**, 83-109 (1989).
149. Westerterp, K. R. Physical activity assessment with accelerometers. *Int J Obes Relat Metab Disord* **23 Suppl 3**, S45-9. (1999).
150. Westerterp, K. R. Pattern and intensity of physical activity. *Nature* **410**, 539. (2001).
151. Larson-Meyer, D. E. et al. Effect of weight reduction, obesity predisposition, and aerobic fitness on skeletal muscle mitochondrial function. *Am J Physiol Endocrinol Metab* **278**, E153-61. (2000).

152. Larson-Meyer, D. E., Newcomer, B. R., Hunter, G. R., Hetherington, H. P. & Weinsier, R. L. 31P MRS measurement of mitochondrial function in skeletal muscle: reliability, force-level sensitivity and relation to whole body maximal oxygen uptake. *NMR Biomed* **13**, 14-27. (2000).
153. Mahler, D. A., Froelicher, V. F., Miller, N. H. & York, T. D. *ACSM's Guidelines for Exercise Testing and Prescription* (eds. Kenney, W. L., Humphrey, R. H. & Bryant, C. X.) (Williams & Wilkins, Media, PA, 1995).
154. Livesey, G. & Elia, M. Estimation of energy expenditure, net carbohydrate utilization, and net fat oxidation and synthesis by indirect calorimetry: evaluation of errors with special reference to the detailed composition of fuels. *Am J Clin Nutr* **47**, 608-28. (1988).
155. Elia, M. & Livesey, G. Theory and validity of indirect calorimetry during net lipid synthesis. *Am J Clin Nutr* **47**, 591-607. (1988).
156. de Jonge, L. & al., e. *Int J Obes* (2001, In Press).
157. Flint, A., Raben, A., Blundell, J. E. & Astrup, A. Reproducibility, power and validity of visual analogue scales in assessment of appetite sensations in single test meal studies. *Int J Obes Relat Metab Disord* **24**, 38-48. (2000).
158. Keim, N. L., Stern, J. S. & Havel, P. J. Relation between circulating leptin concentrations and appetite during a prolonged, moderate energy deficit in women. *Am J Clin Nutr* **68**, 794-801. (1998).
159. Schoeller, D. A. Measurement of energy expenditure in free-living humans by using doubly labeled water. *J Nutr* **118**, 1278-89. (1988).
160. Racette, S. B. et al. Relative dilution spaces of 2H- and 18O-labeled water in humans. *Am J Physiol* **267**, E585-90. (1994).
161. DeLany, J. P., Schoeller, D. A., Hoyt, R. W., Askew, E. W. & Sharp, M. A. Field use of D2 18O to measure energy expenditure of soldiers at different energy intakes. *J Appl Physiol* **67**, 1922-9. (1989).
162. Schulz, L. O., Alger, S., Harper, I., Wilmore, J. H. & Ravussin, E. Energy expenditure of elite female runners measured by respiratory chamber and doubly labeled water. *J Appl Physiol* **72**, 23-8. (1992).
163. Wasserman, K. & Whipp, B. J. Excercise physiology in health and disease. *Am Rev Respir Dis* **112**, 219-49. (1975).
164. Li, H. et al. Quantitative high performance liquid chromatography/tandem mass spectrometric analysis of the four classes of F(2)-isoprostanes in human urine. *Proc Natl Acad Sci U S A* **96**, 13381-6. (1999).
165. Reznick, A. Z. et al. Antiradical effects in L-propionyl carnitine protection of the heart against ischemia-reperfusion injury: the possible role of iron chelation. *Arch Biochem Biophys* **296**, 394-401. (1992).
166. Clauss, A. Rapid physiological coagulation method for the determination of fibrinogen. *Acta Haematol* **17**, 237-46 (1957).
167. Celermajer, D. S., Sorensen, K. E., Bull, C., Robinson, J. & Deanfield, J. E. Endothelium-dependent dilation in the systemic arteries of asymptomatic subjects relates to coronary risk factors and their interaction. *J Am Coll Cardiol* **24**, 1468-74. (1994).
168. Welch, S., Gebhart, S. S., Bergman, R. N. & Phillips, L. S. Minimal model analysis of intravenous glucose tolerance test-derived insulin sensitivity in diabetic subjects. *J Clin Endocrinol Metab* **71**, 1508-18. (1990).
169. Bergman, R. N., Ider, Y. Z., Bowden, C. R. & Cobelli, C. Quantitative estimation of insulin sensitivity. *Am J Physiol* **236**, E667-77. (1979).

170. Lovejoy, J. C. et al. Effects of experimentally induced mild hyperthyroidism on growth hormone and insulin secretion and sex steroid levels in healthy young men. *Metabolism* **46**, 1424-8. (1997).
171. Bjorntorp, P., Holm, G. & Rosmond, R. Hypothalamic arousal, insulin resistance and Type 2 diabetes mellitus. *Diabet Med* **16**, 373-83. (1999).
172. Rosmond, R., Holm, G. & Bjorntorp, P. Food-induced cortisol secretion in relation to anthropometric, metabolic and haemodynamic variables in men. *Int J Obes Relat Metab Disord* **24**, 416-22. (2000).
173. Gao, Y. Y. et al. Autonomic activity assessed by heart rate spectral analysis varies with fat distribution in obese women. *Obes Res* **4**, 55-63. (1996).
174. Tataranni, P. A., Young, J. B., Bogardus, C. & Ravussin, E. A low sympathoadrenal activity is associated with body weight gain and development of central adiposity in Pima Indian men. *Obes Res* **5**, 341-7. (1997).
175. Schena, M. et al. Parallel human genome analysis: microarray-based expression monitoring of 1000 genes. *Proc Natl Acad Sci U S A* **93**, 10614-9 (1996).
176. Alizadeh, A. A. et al. Distinct types of diffuse large B-cell lymphoma identified by gene expression profiling [see comments]. *Nature* **403**, 503-11 (2000).
177. Kononen, J. et al. Tissue microarrays for high-throughput molecular profiling of tumor specimens [see comments]. *Nat Med* **4**, 844-7 (1998).
178. Nides, M. et al. Weight gain as a function of smoking cessation and 2-mg nicotine gum use among middle-aged smokers with mild lung impairment in the first 2 years of the Lung Health Study. *Health Psychol* **13**, 354-61. (1994).
179. Geiselman, P. J., Smith, S. R., DeLany, J. P., Anderson, A. M. & Dowdy, M. L. Reliability of a macronutrient self-selection paradigm in women and validations against erythrocyte membrane fatty acid content and doubly labeled water. (Manuscript in preparation, 2001).
180. Champagne, C. FOODS Validation Study. *Delta Niri Consortium* (Personal Communication).
181. Ravussin, E., Lillioja, S., Anderson, T. E., Christin, L. & Bogardus, C. Determinants of 24-hour energy expenditure in man. Methods and results using a respiratory chamber. *J Clin Invest* **78**, 1568-78 (1986).
182. Cohen, J. in *Statistical Power Analysis for Behavioral Sciences* 277 (Lawrence Erlbaum Assoc., Publishers, Hillsdale, New Jersey, 1988).
