# Supplementary material for: Calorie Restriction Increases Muscle Mitochondrial Biogenesis in Healthy Humans
Source: PLoS Med. 2007 Mar 6;4(3):e76. doi: 10.1371/journal.pmed.0040076 (PMC1808482; doi:10.1371/journal.pmed.0040076)
Supplement: Supplementary file 2 [file pmed.0040076.st001.doc]

**Supplementary Table 1.** Oligonucleotide sequences for primer/probe sets used in Taqman analysis.

| **Gene** | **Sense (5′-′3)** | **Antisense (5′-′3)** | **Probe (5′ 6-FAM-BHQ-1 ′3)** |
| --- | --- | --- | --- |
|  | **Signaling/Transcription** | | |
| AMPK-α2 | aactgcagagagccattcacttt | ggtgaaactgaagacaatgtgctt | tggctctctcactggctctttgaccg |
| SIRT1 | tacgacgaagacgacgacga | cgccgccgccgcctcttcc | aaggttatctcggtacccaatcg |
| PPAR-α | gctttggctttacggaatacca | tgaaagcgtgtccgtgatga | agccatctgagccaggacagcttccttaa |
| PPAR-δ | gtcaaacgagagtcagcctttaacg | ccacggagctgatcccaa | agagatgccattctggcccaccaactt |
|  | **Mitochondrial Oxidation** | | |
| CPT1B | gaggcctcaatgaccagaatg | gtggactcgctggtacaggaa | cagtctcagtccgtccctcccgg |
| CS | ttggctgacctgatacctaagg | caccatacatcatgtccacagtg | ccaccgtcttgccatgttgctgcc |
| COXII | Ttttcaggcttcaccctagatga | gaagaatgttatgtttactcctacgaatatg | catgagcaaaagcccacttcgcca |
|  | **Mitochondrial Fusion** | | |
| PSARL | ccttccgatgttcacgttcac | ccaagatgtgccgcatgat | tgccctgaaagccattatcgccatg |
|  | **Mitochondrial Biogenesis** | | |
| PGC1α | tcctcttcaagatcctgctattac | gacggctgtagggcgatc | aagccactacagacaccgcacgc |
| PGC1β | cagccactcgaaggaacttca | cggatgcttggcgttctg | ctgaacacggccctctgctctcaca |
| NFR1 | cgttgcccaagtgaattattctg | ccctgtaacgtggcccaat | ttgttccacctctccatcagcca |
| mtTFA | cccagatgcaaaaactacagaactaa | tccgccctataagcatcttga | tccaacgctgggcaattcttcta |
| eNOS | ggcatcaccaggaagaacacc | tcactcgcttcgccatcac | ccaacgccgtgaagatctccgc |
|  | **Oxidative Stress** | | |
| HO1 | ccatgacaccaaggaccagag | gggcagaatcttgcactttgtt | tcacgggcaccagggcttcgc |
| SOD2 | gcttgtccaaatcaggatcca | gcgtgctcccacacatca | cccagcagtggaataaggcctgttgtt |
|  | **Internal qPCR control** | | |
| RPLPO | ccattctatcatcaacgggtacaa | agcaagtgggaaggtgtaatcc | tctccacagacaaggccaggactcg |
| **Mitochondrial DNA assay** | | | |
| ND1 | ccctaaaacccgccacatct | gagcgatggtgagagctaaggt | ccatcaccctctacatcaccgccc |
| ND4 | ccattctcctcctatccctcaac | cacaatctgatgttttggttaaactatattt | ccgacatcattaccgggttttcctcttg |
| LPL | cgagtcgtctttctcctgatgat | ttctggattccaatgcttcga | acattcaccagagggtc |

AMPK- α2, AMP-activated protein kinase alpha 2 catalytic subunit**;** CPT1B, carnitine palmitoyltransferase 1B; PGC1α, peroxisome proliferative activated receptor gamma coactivator 1-alpha; PGC1β, peroxisome proliferative activated receptor gamma coactivator 1-beta, NRF1, nuclear respiratory factor 1; mtTFA, mitochondrial transcription factor A; CS, citrate synthase; PPAR-α, peroxisome proliferative activated receptor-alpha; PPAR-δ, peroxisome proliferative activated receptor-delta; HO1, heme oxygenase; PSARL, presenillin-associated rhomboid-like protein; COXII, cytochrome c oxidase subunit 2; SOD2, superoxide dismutase 2; SIRT1, sirtuin 1; eNOS, endothelial nitric oxide synthase; RPLPO, ribosomal protein large, PO: ND1, NADH dehydrogenase subunit 1; ND4, NADH dehydrogenase subunit 4; LPL, lipoprotein lipase.
